# Exploring a panel of serum biomarkers for cancer risk in patients with non-specific symptoms: a comparative analysis of feature selection methods

**DOI:** 10.1136/bmjopen-2025-099967

**Published:** 2025-12-10

**Authors:** Maria Jose Monroy-Iglesias, Aida Santaolalla, Sabine Martin, Bernard North, Charlotte Moss, Kate Haire, Geraint Jones, Lindsay Steward, Carlos Cargaleiro, Flaminia Bruno, Juliet Millwaters, Chandra Basyal, Sarah Weild, Beth Russell, Mieke Van Hemelrijck, Saoirse Dolly

**Affiliations:** 1 Transforming Cancer Research Outcomes Through Research (TOUR), King’s College London, London, UK; 2 Medical Oncology, Guy’s and St Thomas’ NHS Foundation Trust, London, England, UK; 3 Cancer Prevention Trials Unit, Cancer Prevention Group, King’s College London, London, UK; 4 South East London Cancer Alliance, London, England, UK; 5 Specialist Medicine, Queen Elizabeth Hospital, Lewisham & Greenwich NHS Trust, London, England, UK

**Keywords:** EPIDEMIOLOGY, ONCOLOGY, Primary Care

## Abstract

**Abstract:**

**Objectives:**

Delays in cancer diagnosis for patients with non-specific symptoms (NSSs) lead to poorer outcomes. Rapid Diagnostic Clinics (RDCs) expedite care, but most NSS patients do not have cancer, highlighting the need for better risk stratification. This study aimed to develop biomarker-based clinical prediction scores to differentiate high-risk and low-risk NSS patients, enabling more targeted diagnostics.

**Design:**

Retrospective and prospective cohort study.

**Setting:**

Secondary care RDC in London.

**Participants:**

Adult patients attending an RDC between December 2016 and September 2023 were included. External validation used data from another RDC.

**Outcome measures:**

The primary outcome was a cancer diagnosis. Biomarker-based risk scores were developed using Latent Class Analysis (LCA) and Least Absolute Shrinkage and Selection Operator (LASSO). Model performance was assessed using logistic regression, receiver operating characteristic curves (AUROC) and decision curve analysis.

**Results:**

Among 5821 RDC patients, LCA identified high white cell count, low haemoglobin, low albumin, high serum lambda light chain, high neutrophil-to-lymphocyte ratio, high serum kappa light chain (SKLC), high erythrocyte sedimentation rate (ESR), high C-reactive protein (CRP) and high neutrophils as cancer risk markers. LASSO selected high platelets, ESR, CRP, SKLC, alkaline phosphatase and lactate dehydrogenase. Each one-point increase in score predicted higher odds of cancer (LCA: AOR 1.19, 95% CI 1.16 to 1.23; LASSO: AOR 1.29, 95% CI 1.25 to 1.34). Scores ≥2 predicted significantly higher cancer odds (LCA: AOR 3.79, 95% CI 2.91 to 4.95; LASSO: AOR 3.44, 95% CI 2.66 to 4.44). Discrimination was good (AUROC: LCA 0.74; LASSO 0.73). External validation in 573 patients confirmed predicted increases in cancer risk per one-point LASSO score rise (AOR 1.28, 95% CI 1.15 to 1.42), with a borderline increase for LCA (AOR 1.16, 95% CI 1.06 to 1.27).

**Conclusion:**

Biomarker-based scores effectively identified NSS patients at higher cancer risk. LCA captured a broader biomarker range, offering higher sensitivity, while LASSO achieved higher specificity with fewer markers. These scores may also help detect severe benign conditions, improving RDC triage. Further validation is needed before broader clinical implementation.

**STRENGTHS AND LIMITATIONS OF THIS STUDY**

- Large, reliable study population with detailed clinical and sociodemographic data.
- High-quality prospective data, allowing for robust analysis and replication in primary care.
- Comprehensive evaluation of non-specific symptoms, beyond just weight loss, addressing gaps in existing literature.
- Missing data on several biomarkers limits imputation techniques and could introduce bias.
- Generalisability of identified biomarker combinations may be limited to specific patient populations.

## Introduction

 Non-site-specific (NSS) symptoms, such as fatigue, unexplained weight loss or progressive pain, often pose a diagnostic challenge for clinicians who must distinguish between benign and malignant conditions.^1^ It has been reported that malignancies presenting with NSS symptoms are often diagnosed at a later stage with higher mortality rates.^2 3^ This highlights the need to streamline current cancer pathways and improve diagnostic strategies to facilitate earlier detection and intervention for these NSS patients. As such, rapid diagnostic clinics (RDCs) were established in England to provide a streamlined NSS pathway by prioritising diagnostics, while also managing patients’ comorbidities, polypharmacy, nutrition and mental health conditions.^4^

At the RDC at Guy’s and St Thomas’ NHS Foundation Trust (Guy’s RDC), a multidisciplinary team including oncology, acute medicine and general practice consultants conducts comprehensive assessments during initial consultations. These assessments cover symptoms, physical and mental health, lifestyle, social factors and nutrition, alongside physical examinations. Patients also provide biological samples (blood and urine) and undergo diagnostic tests within 2 weeks to ensure timely diagnoses and arrange specialist evaluations if needed, or to return patients to their general practitioner (GP) when appropriate. A previous analysis of the Guy’s RDC cohort reported that 7% of patients were diagnosed with cancer, with 31% at an early stage, and 36% had serious benign conditions warranting onward referral. This confirmed that RDCs offer a more efficient pathway for complex NSS patients.^3^ However, despite the usefulness of these clinics, most patients with NSS symptoms do not have a serious underlying cause.^3 5^ This suggests the need for diagnostic strategies that can differentiate between patients requiring urgent testing and those with a lower risk of cancer who may benefit from a watchful waiting approach.^6^ Such a strategy would help prevent low-risk patients from undergoing unnecessary tests, thereby reducing potential patient anxiety and healthcare costs.

To address these diagnostic challenges, various studies have explored biomarker-based prediction models for patients presenting with NSS symptoms across primary and secondary care settings.^7^,^10^ For instance, Hernandez *et al* developed a clinical prediction score incorporating age (≥80 years), serum albumin, white cell count (WCC), alkaline phosphatase (ALP) and lactate dehydrogenase (LDH), which allowed for the correct classification of cancer versus non-cancer cases in 85% of the patients included in their analysis, with an area under the curve (AUC) of 0.90. However, a subsequent study validating this model using secondary care data showed lower accuracy (AUC of 0.69), suggesting this model may have limitations. Moreover, a recent UK-based study aimed at identifying clinical features to stratify patients presenting with weight loss in primary care found that a combination of basic blood test abnormalities could effectively identify patients warranting further investigation.^9^ Notably, the blood tests included in this model were WCC, ALP, albumin (similar to the model developed by Hernandez *et al*), liver enzymes, C-reactive protein (CRP), haemoglobin (Hb) and platelets. While these studies have predominantly focused on patients presenting with unintentional weight loss, there remains a gap in understanding NSS populations which encompass a broader range of symptoms such as fatigue and progressive pain.

The current study aimed to develop two new clinical prediction scores using two distinct statistical approaches for feature selection. Based on basic serum biomarker panels undertaken at first outpatient consultation at Guy’s RDC, these scores aimed to categorise patients into low and high-risk groups. By doing so, they may enable expedited diagnostic testing for high-risk patients while permitting low-risk patients to adopt a watchful waiting approach.

## Methods

### Study setting and biomarker ascertainment

Our study population comprised adult patients who attended Guy’s RDC in Southeast London, between December 2016 and September 2023. Patients are typically referred to the RDC by their GP when presenting with NSS symptoms, such as unexplained weight loss, fatigue or persistent pain, that may be suggestive of an underlying malignancy but do not immediately point to a specific cancer site.

At the time of their first RDC visit, patients undergo a comprehensive set of blood tests during their initial consultation at the RDC.^4 11^ The tests vary according to clinicians’ judgement but typically include a range of serum biomarkers such as: WCC, Hb, platelets, neutrophils, lymphocytes, calcium, bilirubin, ALP, alanine transaminase (ALT), albumin, creatinine, LDH, CRP, ferritin, erythrocyte sedimentation rate (ESR), serum Kappa light chains (SKLCs), serum Lambda light chains (SLLCs) and estimated glomerular filtration rate (eGFR). All samples were processed at the GSTT clinical biochemistry laboratory following standard hospital procedures. Serum and plasma were analysed on the same day as collection using automated platforms in line with manufacturer protocols. Internal and external quality assurance schemes are routinely followed within the hospital laboratory. Limits of detection and assay-specific details are provided in online supplemental Table 1).

Clinical and sociodemographic data were collected retrospectively from the electronic patient records for patients seen between December 2016 and May 2020. For patients seen from June 2020 onwards, data were collected prospectively and managed using REDCap electronic data capture tools hosted at GSTT.^12^ Variables included age, sex, ethnicity and socioeconomic status, the latter obtained by linking patient postcodes to the Index of Multiple Deprivation using publicly available data from the Ministry of Housing, Communities and Local Government (2019). Lastly, patients who opted out of the NHS Digital National data opt-out scheme were excluded from analysis.^13^ All data were collected and analysed under the ethical approval of GSTT Cancer Cohort (Ethics reference number: 23/NW/0105), which permits the use of data collected via the NHS Opt-Out Scheme, thus negating the need for individual patient consent.

### Outcome ascertainment

The primary outcome of interest was a confirmed cancer diagnosis. Guy’s RDC aims to provide a diagnosis within a few weeks of referral (median 28 days, Reference). Patients with suspicious imaging or clinical findings are referred to urgent site-specific pathways for confirmation, while others are discharged once malignancy has been excluded. Outcome data were collected until a formal diagnosis was recorded or the patient was discharged.

### Statistical analysis

**Figure 1:**
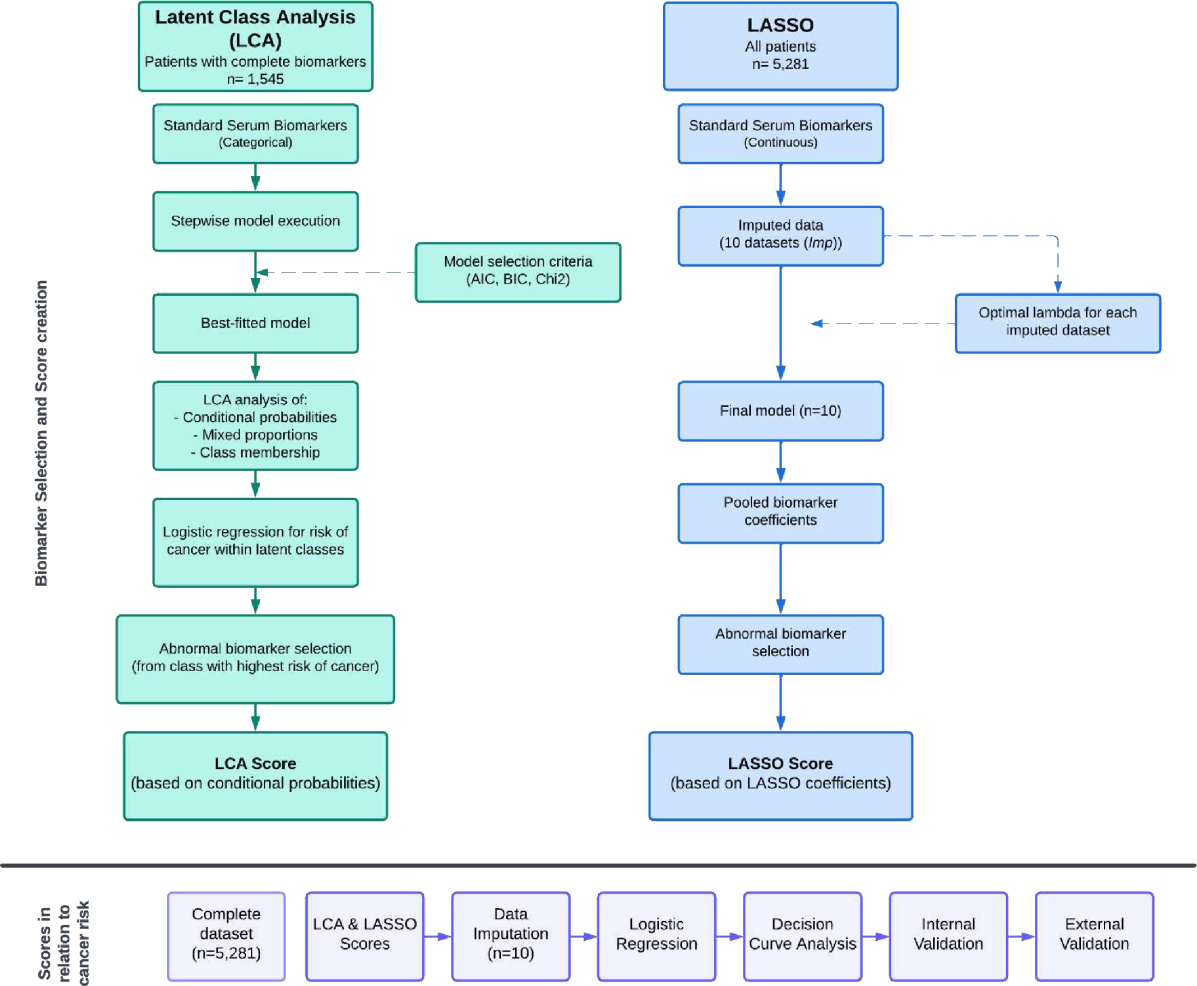
Model construction and evaluation steps. AIC, Akaike information criterion; BIC, Bayesian information criterion; Chi2, χ^2^ distribution; LCA, latent class analysis; LASSO, least absolute shrinkage and selection operator.

Two feature selection analyses were conducted to create biomarker-based risk scores: latent class analysis (LCA) and least absolute shrinkage and selection operator (LASSO). Using both methods allows for a comparison between supervised (with outcome data) and unsupervised models (without outcome data), helping to evaluate the effectiveness of each approach. Figure 1 summarises the model construction and data analysis steps.

Our analyses primarily focused on the previously mentioned biomarkers. In addition, we derived additional ratios from the following biomarkers: neutrophil to lymphocyte ratio (NLR) from the results of lymphocytes and neutrophils, and serum free light chain ratio (SFLC) from SKLC and SLLC.^14 15^ Biomarkers included in LCA were categorised using standardised clinical cut-offs established by the laboratory normal ranges to facilitate model convergence and interpretability. A detailed breakdown of the specific levels for each biomarker is provided in online supplemental Table 2. For the LASSO analyses, these biomarkers were treated as continuous variables to preserve the full range of information and ensure stable coefficient estimation. The primary outcome was diagnosis of cancer of any type usually identified histologically. In a minority of cases, if the patients were not well enough for biopsy, these were confirmed radiologically.

For our descriptive statistics, categorical variables are presented as counts and percentages, while continuous variables are reported as means with SD or medians with IQRs. We compared sociodemographic, clinical and biomarker characteristics between cancer and non-cancer patients using statistical tests, such as Student’s t-test, Pearson’s χ^2^ test or the Wilcoxon rank sum test, depending on the data type and distribution.

Moreover, before running our two feature selection methods, we calculated Pearson correlation coefficients to evaluate associations among the continuous biomarker variables in our analysis. High correlations were observed between lymphocytes, leukocytes, NLR and renal biomarkers (creatinine and eGFR). To mitigate collinearity and adhere to the principle of local independence, we excluded lymphocytes and eGFR from further analyses.

#### Latent class analysis

We performed LCA, a model-based clustering method that groups covariates into latent classes, capturing underlying unobservable conditions such as potential cancer.^16^ Given the criteria of LCA, which requires complete data on all included variables (in this case, biomarkers), we used a subset of patients with complete biomarker data for this analysis. Imputed values were not used, as standard imputation methods do not preserve the latent structure underlying LCA and could bias class formation. As previously mentioned, we used categorical variables based on standardised clinical cut-offs in our LCA.

To find the optimal number of LCA-derived classes, we started with the null model and added classes iteratively up to the number of biomarkers (n=19). Model selection criteria such as Akaike Information Criterion (AIC), Bayesian Information Criterion (BIC) and χ^2^ distribution guided us to the best-fitting model. When plotting these metrics (online supplemental figure 1), the χ^2^ curve provided the clearest indication of the optimal model, with a distinct drop and lowest value at three classes. AIC and BIC showed a smaller but consistent change at the same point, further supporting the three-class solution. To understand which biomarker sets predominantly explained each latent class, how classes distributed across the study population, and which individuals belonged to each class, we examined conditional probabilities, mixed proportions and class memberships of the best-fitted latent class model.

Subsequently, each subject was assigned to a latent class, and a binary logistic regression was used to ORs and 95% CIs for overall cancer risk. We used a latent class with a potential benign diagnosis as the reference group. The class with the highest OR for cancer risk underwent further variable selection. Biomarkers with conditional-item response probabilities exceeding 0.40 were included in forming the LCA score. Contributions to the score were determined by each variable’s conditional-item response probability within the selected class. Points were assigned based on probabilities: 0.40–0.60: 1 point, 0.61–0.80: 2 points, >0.81: 3 points.

#### Missing data

Among the predictor variables, the proportion of missing data ranged from 0% to 53% (online supplemental Table 3). Missing biomarker data were handled using multiple imputation by chained equations (MICE) in R (package mice) under the ‘missing at random’ assumption. Predictive mean matching was applied for continuous variables and logistic regression for binary variables, with 20 iterations per imputation cycle. Two imputation models were created, each generating ten imputed datasets and including all predictor variables, endpoint indicators (ie, cancer diagnosis), sex and age. These imputed datasets were used for subsequent analyses, including LASSO selection and logistic regression, while LCA was performed solely for variable selection using complete cases.

#### LASSO

LASSO regression performs variable selection and regularisation by shrinking coefficients, allowing the identification of significant predictors and exclusion of less important ones. We included standardised continuous biomarker variables in the analysis, using overall cancer diagnosis as the outcome variable. We conducted 10-fold cross-validation on 10 imputed datasets to determine the optimal penalty factor (λ). The mean λ across datasets was calculated for subsequent analysis. Using this mean λ, we fitted a LASSO model in each imputed dataset and pooled the coefficients. Significant variables were those with non-zero coefficients, and they were incorporated into a second cancer risk score called the LASSO score. Variables were scored based on their coefficients: 0–0.10: 1 point, 0.11–0.20: 2 points, >0.21: 3 points.

#### Logistic regression for scores

Once the LCA and LASSO scores were derived, they were applied to each imputed dataset and analysed as continuous and binary variables, both per one-point increase and per one SD increase, and as binary variables, using a cut-off determined based on the score distribution in the overall population. Binary logistic regression was then employed to assess the association between these scores and overall cancer risk. Both unadjusted and adjusted models were considered, with sex and age as covariates. Rubin’s rules were used to combine estimates and standard errors across imputations, resulting in a pooled model for predictor selection.

#### Internal cross-validation

Ten-fold internal cross-validation was used to assess performance for all logistic regression models. The discriminative abilities of the scores were assessed and compared using the area under the receiver operating characteristic curve (AUROC), and 95% CIs were calculated using bootstrap resampling.

#### Decision curve analysis

We conducted decision curve analysis (DCA) to assess the clinical utility of the binary LCA and LASSO models compared with scenarios where no prediction model was employed (ie, treat all or treat none). DCA evaluates the net benefit, representing the minimum probability of disease at which further intervention should be warranted, across the specific risk thresholds (in this study, 0% to 15%). The model with the highest net benefit at any given risk threshold is deemed to have the greatest clinical utility. Additionally, we calculated interventions avoided, indicating the proportion of patients who would forego further investigation or intervention without missing a cancer diagnosis at each risk threshold.

#### External validation

An external validation was conducted using data from the recently established Queen Elizabeth Hospital RDC (QEH RDC), also located in southeast London. Established in September 2022 following the model of Guy’s RDC, it operates under the same referral pathways, with biomarker collection at the initial visit. The centre’s multidisciplinary team includes GPs, advanced clinical practitioners and psychology specialists. Data collection at both sites was standardised using REDCap to ensure consistency. The validation process involved applying the previously developed LCA and LASSO scores to the external dataset, considering them as both continuous (single-point and SD increase) and binary variables. This included imputation (MICE with the same parameters as the main analysis), logistic regression to assess the association between the scores and the risk of overall cancer.

Data management and statistical analyses were performed using Stata 18 (Stata, College Station, Texas, USA) and R V.4.0.1 (mice, poLCA, glmnet, pROC, dcurves packages). Our manuscript is in line with the Transparent reporting of a multivariable prediction model for individual prognosis or diagnosis (TRIPOD) reporting guidelines for prediction models (online supplemental Table 4).

## Results

### Characteristics of the study population

A total of 5821 patients attended Guy’s RDC between December 2016 and September 2023. Of these, 6.3% (n=364) were diagnosed with a cancer, 32.5% (n=1892) with a serious benign condition which was defined by an onward referral to a specialist, 29.1% (n=1694) were sent back to their GP with a non-serious benign condition and lastly, 32.1% (n=1871) of patients were sent back to their GP with no further diagnosis. (online supplemental Table 5) presents patient sociodemographic and biomarker characteristics overall and by cancer status. Among cancer patients, 55% were male compared with 40% in the non-cancer group. Cancer patients were also older, with a mean age of 68 years (SD 13), compared with 61 years (SD 15) for non-cancer patients. Both groups showed similar distributions in terms of ethnicity and socioeconomic status.

A subset of 1545 patients with complete biomarker data was analysed in our LCA. This subset closely resembled the full study population in terms of sociodemographic and clinical characteristics, as shown in online supplemental Table 6. Within this group, 6.6% (n=102) were diagnosed with cancer, 33% (n=510) had a serious benign condition, 36.3% (n=561) were referred back to their GP with a non-serious benign condition, and 24.1% (n=372) were referred back to their GP without a further diagnosis.

### LCA score

Model selection criteria identified the optimal model with three latent classes. The three LCA-derived classes with their respective population proportions and class conditional probabilities for each biomarker are outlined in table 1:

1. Class 1—‘Normal’ (61%): this group had low probabilities (<0.4) for all abnormal values of biomarkers, representing the general population without significant health concerns.
2. Class 2—‘Serious condition’ (32%): this group had high probabilities for abnormal values of Hb (low), albumin (low), SKLC (high), SLLC (high), ESR (high) and CRP (high), suggesting a potential serious condition.
3. Class 3—‘Very serious condition’ (7%): this group had high probabilities for abnormal values of WCC (high), Hb (low), NLR (high), neutrophils (high), albumin (low), SKLC (high), SLLF (high), ESR (high) and CRP (high), suggesting a possible very serious condition.

**Table 1:** Predicted class memberships of the clinically abnormal biomarker cut-off values for the three latent classes model, estimated class population shares for the latent classes and conditional item response probabilities

Latent class analysis-derived classes	Class 1	Class 2	Class 3
	‘Normal’	‘Serious condition’	‘Very serious condition’
Estimated class proportions (% of population)	*61%*	*32%*	*7%*
*High white cell count*	0.0085	0	**0.5389**
*Low haemoglobin*	0.1506	**0.5067**	**0.4105**
*High platelets*	0.0186	0.0714	0.3326
*High Neutrophil-to-lymphocyte ratio*	0.0983	0.1894	**0.6697**
*High Neutrophils*	0.0288	0.0095	**0.8082**
*High Calcium*	0.0571	0.1258	0.1777
*High Creatinine*	0.1944	0.3275	0.2143
*High Bilirubin*	0.0386	0.0537	0.0782
*High Alanine Transaminase*	0.0912	0.0968	0.0895
*High Alkaline Phosphatase*	0.0284	0.1893	0.2844
*Low Albumin*	0.1005	**0.4608**	**0.4192**
*High Lactate Dehydrogenase*	0.2484	0.38	0.322
*High Serum Kappa Light Chain*	0.3951	**0.9584**	**0.7473**
*High Serum Lambda Light Chain*	0.0143	**0.5138**	**0.4027**
*High Serum Free Light Chain Ratio*	0.1235	0.3055	0.096
*High Ferritin*	0.1064	0.2214	0.3786
*High Erythrocyte Sedimentation Rate*	0.1446	**0.7661**	**0.7235**
*High C-Reactive Protein*	0.1183	**0.4916**	**0.7753**

High probabilities of the biomarkers belonging to a class (≥0.4) are highlighted.

When assessing cancer risk, both Class 3 (‘very serious condition’) and Class 2 (‘serious condition’) predicted significantly higher odds of cancer diagnosis (OR 4.26; 95% CI 2.26 to 8.02 and OR 2.88; 95% CI 1.78 to 4.45, respectively) compared with the reference Class 1 (‘Normal’) (online supplemental Table 7). In addition, when examining the final diagnoses by class, it was shown that 15% of individuals in Class 3 had a malignant condition, compared with 10.3% in Class 2 (online supplemental Table 8). Therefore, Class 3 was chosen for variable selection of biomarkers due to its higher cancer risk.

**Figure 2:**
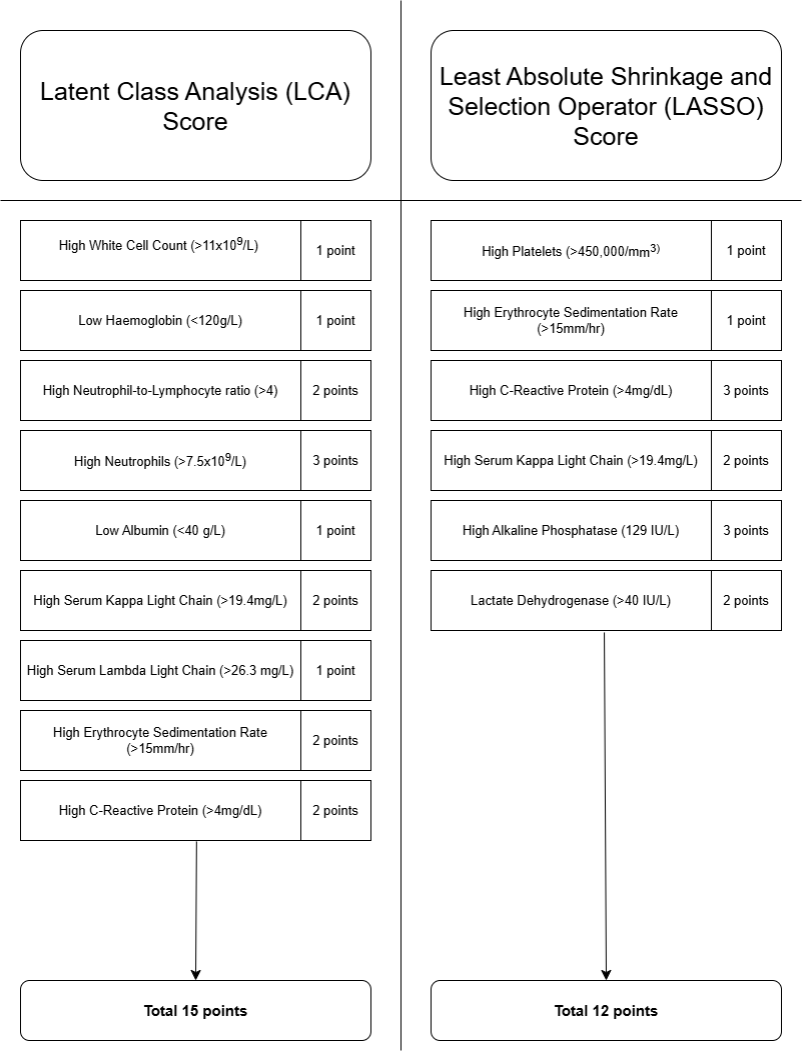
Latent class analysis and least absolute shrinkage and selection operator scores.

Based on conditional-item response probabilities, abnormal biomarkers contributing to the LCA score were identified and scored as follows: 1 point was assigned to high WCC, low Hb, low albumin and high SLLC; two points were assigned to high NLR, high SKLC, high ESR and high CRP and high neutrophil levels were assigned three points. For the full LCA point system, refer to figure 2.

### LASSO score

LASSO regression analysis was used to identify significant predictors of overall cancer risk. Biomarkers with LASSO coefficients above zero were included in the LASSO score and scored according to their significance. The LASSO coefficients for each biomarker are presented in table 2. Significant biomarkers identified and their corresponding point assignments in the LASSO score were as follows: high platelets and high ESR received one point; high SKLC and high LDH received two points and high CRP and high ALP were assigned three points. For the full LASSO point system, refer to figure 2.

**Table 2:** Least absolute shrinkage and selection operator regression coefficients of the continuous biomarker variables

Biomarker	LASSO coefficients
Platelets	***0.0007670892***
Erythrocyte sedimentation rate	***0.0709207062***
C-reactive protein	***0.2330810041***
Serum kappa light chain	***0.1711941691***
Alkaline phosphatase	***0.2703203913***
Lactate dehydrogenase	***0.1644775873***
White cell count	.
Haemoglobin	.
Neutrophils	.
Neutrophil to lymphocyte ratio	.
Ferritin	.
Serum Lambda light chain	.
Serum light chain ratio	.
Creatinine	.
Corrected calcium	.
Bilirubin	.
ALT	.
Albumin	.

Significant (non-zero) regression coefficients of the biomarkers are highlighted.

ALT, alanine transaminase; LASSO, least absolute shrinkage and selection operator.

### Logistic regression

The results of adjusted logistic regression analyses for both the LCA and LASSO scores as continuous variables demonstrated strong predictive ability for cancer. For every one-point increase in score, the odds of a cancer diagnosis increased by 1.19 (adjusted OR 1.19; 95% CI 1.16 to 1.23) for the LCA score and 1.29 (adjusted OR 1.29; 95% CI 1.25 to 1.34) for the LASSO score. Additionally, for every one SD increase in the LCA score, the odds of being diagnosed with cancer were 1.71 times higher (adjusted OR 1.71; 95% CI 1.57 to 1.87), while a one SD increase in the LASSO score corresponded to 1.93 times higher odds (adjusted OR 1.93; 95% CI 1.76 to 2.11) (online supplemental Table 9).

When using binary versions of both scores, a cut-off of 2 was selected based on the mean distribution of each score in the overall population (online supplemental figure 2). Individuals with scores≥2 were more likely to have a cancer diagnosis predicted by both models: LCA (adjusted OR 3.79; 95% CI 2.91 to 4.95) and LASSO (adjusted OR 3.44; 95% CI 2.66 to 4.44) (table 3).

**Table 3:** Results from logistic regression models

Score		Unadjusted	Adjusted*	External validation	External validation adjusted*
		OR (95% CI)	OR (95% CI)	OR (95% CI)	OR (95% CI)
**Latent class analysis scores**					
Continuous		1.21 (1.18 to 1.25)	1.19 (1.16 to 1.23)	1.19 (1.08 to 1.30)	1.16 (1.06 to 1.27)
Binary	<2	1 (Ref)	1 (Ref)	1 (Ref)	1 (Ref)
	>=2	4.19 (3.21 to 5.43)	3.79 (2.91 to 4.95)	2.29 (1.16 to 4.71)	1.84 (0.92 to 3.86)
**LASSO scores**					
Continuous		1.30 (1.26 to 1.35)	1.29 (1.25 to 1.34)	1.30 (1.17 to 1.43)	1.28 (1.15 to 1.42)
Binary	<2	1 (Ref)	1 (Ref)	1 (Ref)	1 (Ref)
	>=2	3.79 (2.95 to 4.0)	3.44 (2.66 to 4.44)	2.50 (1.26 to 5.24)	2.07 (1.02 to 4.40)

* Adjusted for sex and age.

### Internal validation

The binary LCA score demonstrated discrimination with an AUROC of 0.74 (95% CI 0.72 to 0.75). Similarly, the binary LASSO score exhibited discrimination with an AUROC of 0.73 (95% CI 0.71 to 0.74). The binary LCA score had a specificity of 0.57 and sensitivity of 0.82. While the binary LASSO score had a specificity of 0.71 and a sensitivity of 0.67 (table 4).

**Table 4:** Results from internal cross-validation

	AUROC (95% CI)	Specificity	Sensitivity
Latent class analysis*	0.74 (0.72 to 0.75)	0.57	0.82
LASSO regression*	0.73 (0.71 to 0.74)	0.71	0.67

* Adjusted for sex and age.

AUROC, area under the receiver operator curve; LASSO, least absolute shrinkage and selection operator.

### Decision curve analysis

**Figure 3:**
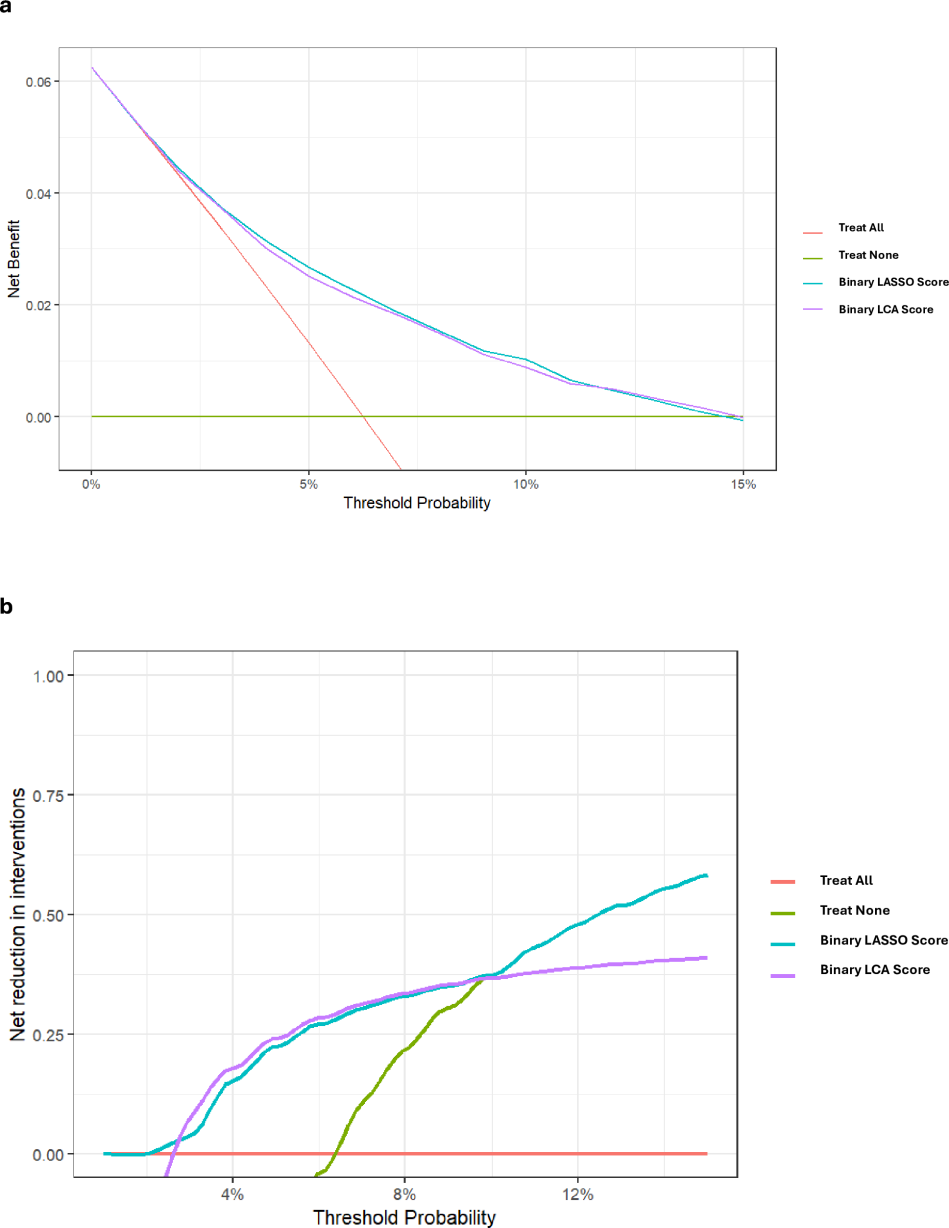
Decision curve analysis: (a) Net benefit and (**b**) Net reductions in interventions. LASSO, least absolute shrinkage and selection operator; LCA, latent class analysis.

The LCA model demonstrated nearly identical clinical utility to the LASSO model (figure 3). Both models exhibited a higher net benefit compared with the ‘investigate all’ approach for cancer risk thresholds from 4% onwards (online supplemental Table 10). Similarly, in terms of interventions avoided, both LCA and LASSO models demonstrated almost identical curves, which were superior to the ‘investigate all’ approach from a threshold of 2%. For instance, at a probability threshold of 6% the net reduction in interventions is about 30% (figure 3).

### External validation

A total of 573 patients attended QEH RDC between September 2022 and January 2024. Of these, 6.6% (n=38) were diagnosed with a cancer. Patient clinical and sociodemographic characteristics overall and by cancer status are detailed in online supplemental Table 11. Gender was comparable between both groups. The mean age was 63 years (SD 16) in the non-cancer group and 71 years (SD 13) in the cancer group. Regarding ethnicity, 86.7% (n=33) of cancer patients were White compared with 70.8% (n=379) of non-cancer patients. In terms of IMD, 42.1% (n=16) of cancer patients had a low IMD, while 33.3% (n=178) of non-cancer patients were in the low IMD category.

For the LCA score, each one-point increase predicted borderline higher odds of cancer (OR 1.16; 95% CI 1.06 to 1.27), while for the LASSO score, each one-point increase predicted significantly higher odds (OR 1.28; 95% CI 1.15 to 1.42). Additionally, for every one SD increase, the odds of cancer were higher for both the LCA score (OR 1.56; 95% CI 1.18 to 2.05) and for the LASSO score (OR 1.98; 95% CI 1.49 to 2.63). When using binary versions of the scores, individuals with a score of 2 or higher were more likely to have a cancer diagnosis predicted by both models. For the LCA score, this association was observed in the unadjusted model (OR 2.29; 95% CI 1.16 to 4.71) but was not retained after adjusting for sex and age. Similarly, for the binary LASSO score, higher predicted odds were seen in the unadjusted model (OR 2.50; 95% CI 1.26 to 5.24), which became borderline significant after adjusting for sex and age (OR 2.07; 95% CI 1.02 to 4.40) (table 3).

## Discussion

In our study of patients attending Guy’s RDC, we developed two clinical prediction scores that effectively stratified patients with NSS symptoms into low and high risk of cancer. For the LCA score, each one-point increase predicted a 19% higher odds of cancer (OR 1.19, 95% CI 1.16 to 1.23), while a cut-off of ≥2 predicted 3.79-fold higher odds (95% CI 2.91 to 4.95). For the LASSO score, each one-point increase predicted a 29% higher odds (OR 1.29, 95% CI 1.25 to 1.34), with a cut-off of ≥2 predicting 3.44-fold higher odds (95% CI 2.66 to 4.44). Both scores highlighted the importance of inflammatory markers such as ESR, CRP and SKLC in cancer prediction, underscoring their potential as key indicators in assessing cancer risk among patients with NSS symptoms. The LCA score encompassed a wider range of markers, such as high WCC, low Hb, high NLR, high neutrophils, low albumin and high SLLC, making it a more sensitive but less specific tool. In contrast, the LASSO score focused on high platelets, ALP and LDH, achieving higher specificity. External validation supported both scores as continuous variables.

### Biomarker tools in other populations of patients with NSS symptoms

Only a handful of studies have looked at the diagnostic value of routine blood tests in association with cancer in patients presenting with NSS symptoms. A Danish study by Naeser *et al* analysed the diagnostic value of an extensive triage blood panel among patients with NSS symptoms referred to a diagnostic centre, resembling the setting of England’s RDCs. This blood panel comprised 27 laboratory tests for men and 26 laboratory tests for women, including both non-cancer specific tests and tumour markers. Notably, the study identified that six or more abnormal blood tests increased the probability of cancer, while fewer than two tests lowered it. Within this study, the most frequent abnormal biomarkers among cancer patients were high inflammatory markers (CRP or ESR), low Hb, low albumin, low lymphocyte count and high ALP.^10^ Furthermore, a previously mentioned study by Hernandez *et al* developed a clinical prediction score based on age (>80 years), low serum albumin, high WCC, high ALP and high LDH, which allowed the correct classification of patients presenting with unexplained weight loss in relation to cancer risk (AUC 0.90).^7^ However, external validation showed a lower AUC (0.69), indicating potential limitations in external validation. In this validation study, prediction power improved by lowering the age cut-off to 60 and removing WCC and high LDH levels.^8^ Lastly, a recent large-scale study examining primary care records of UK patients with unexplained weight loss developed three clinical prediction models, incorporating symptoms alone, symptoms and blood tests, and blood tests only, to evaluate cancer risk. This study by Nicholson *et al* demonstrated that combinations of basic blood test abnormalities, such as low albumin, high ALP, high CRP, low Hb, elevated liver enzymes, increased platelets and elevated WCC, were effective in identifying patients warranting further investigation.^9^ Our study’s findings mostly align with the results of these studies. Both of our scoring systems highlighted and incorporated inflammatory markers such as ESR and CRP, consistent with all previous studies. Moreover, several other biomarkers reported in prior research, including ALP, low Hb, platelets, WCC and albumin, were also identified by at least one of our methods. However, our analysis revealed additional significant biomarkers such as SKLC, neutrophils and NLR, which have not been extensively explored in previous studies. Of these newly described biomarkers associated with cancer in our study, NLR and neutrophils have increasingly been used independently as biomarkers for cancer risk and prognostic indicators for various solid tumours such as breast, lung and colorectal cancers, which reflects the link with the systemic response to a developing cancer.^14 17 18^ On the other hand, SKLC has received comparatively less attention outside the field of multiple myeloma.^15^ A recent review linked altered free light chain levels, including SKLC, to diseases associated with inflammation and immune reactions, which may in turn lead to cancer.^19^ Free light chains have been reported to activate mast cells, leading to pro-tumourigenic effects such as angiogenesis stimulation, extracellular matrix degradation and immunosuppression reactions through the secretion of inflammatory mediators.^20^ Thus, it is reasonable that our study identified these biomarkers, given the diverse range of cancer types diagnosed by our RDC.^3^

### LCA and LASSO score comparison

In comparing both scoring systems developed in this study, it is notable that both scores include markers of inflammation (ie, ESR, CRP and SKLC). However, the differences in the biomarkers included in each score are significant. LCA, an unsupervised method, incorporates a broad range of biomarkers associated with various conditions (ie, cancer and non-cancer). Unlike supervised methods, LCA does not include the outcome variable (in our case, cancer) as a predictor. Consequently, the LCA groups are formed based on latent variables, indicating that the resulting score is tailored to our specific population. This tailored nature of LCA may lead to latent classes that predominantly consist of cancers but also possibly include some serious benign conditions. For instance, abnormal NLR and neutrophils emerged as robust biomarkers within our LCA score. As previously mentioned, these biomarkers have increasingly been recognised as indicators of cancer risk. However, they are also associated with severe infections and other non-cancerous inflammatory conditions.^21^ In contrast, LASSO is a supervised model, meaning that cancer is included in the model as an outcome, and thus, the results are likely tailored to the cancer diagnoses in our NSS patient population. It is also important to note that part of the difference in biomarkers identified by LCA versus LASSO reflects how the data were handled: LCA used binary biomarkers based on clinical cut-offs, whereas LASSO used continuous biomarkers, preserving the full range of information. A previously published study looking at patients seen at Guy’s RDC reported that 40% of patients were diagnosed with a metastatic malignancy.^3^ In addition, a recent unpublished evaluation of RDCs in southeast London reported that over 63% of cancer diagnoses at Guy’s RDC were diagnosed at a late stage (stages 3 and 4). Consequently, the biomarkers selected by LASSO are likely associated with late-stage cancers. For instance, ALP emerged as the biomarker of highest significance within our LASSO model. Recent literature looking at ALP in the context of cancer has linked elevated levels to bone metastases in various cancers, including prostate, breast and other types, underscoring its potential as a marker for advanced disease.^22^ Moreover, the observed high sensitivity in LCA, alongside its broader inclusion of biomarkers, suggests its ability to detect a wide range of conditions, including both cancers and serious benign conditions. Within the LCA, some biomarkers—such as WCC and neutrophils—were elevated in both Class 2 and Class 3, reflecting the continuum of serious conditions captured by this unsupervised method. Class 3 was enriched for malignancy, supporting its prioritisation for biomarker selection to focus on features most indicative of cancer, while the overlap with Class 2 does not compromise the clinical utility of the score. In contrast, the LASSO score exhibits higher specificity but lower sensitivity, likely due to its focus on specific cancer diagnoses within our population. In the clinical context of NSS services, prioritising sensitivity (as demonstrated by LCA) is crucial, ensuring that potential cancer cases are not missed, even if it requires investigating non-cancer patients.

### Clinical considerations

Lastly, it is crucial to acknowledge that while our blood panels demonstrated strong discrimination between patients with and without cancer, they may not suffice as standalone diagnostic tools in clinical decision-making. The consideration of clinical and sociodemographic patient characteristics remains paramount, and blood panel results could provide additional information to decide on the need for further diagnostics. Previous research has emphasised the importance of incorporating age, sex, smoking history and concurrent clinical features to determine the risk of cancer, and in turn, to justify invasive investigations in the NSS population.^23^

The clinical prediction scores developed in this study address critical challenges in current practice. Traditional assessment methods may overlook patients with subtle or atypical presentations, leading to a longer diagnostic interval, diagnoses or unnecessary anxiety for those with benign conditions. By stratifying patients based on risk levels, these scores enable tailored management strategies, ensuring timely investigations for high-risk individuals while minimising unnecessary testing for those deemed low risk. Additionally, a risk-based model may promote uniformity across NSS services, offering a standardised approach that could replicate successful outcomes. Currently, different services operate independently, which can lead to inconsistencies in patient management. By establishing a common framework, the risk-based model encourages best practices and improves overall quality of care. Integrating these prediction scores into clinical decision-making could enhance diagnostic pathways. Clinicians could use the scores to guide discussions about the necessity for further testing or referrals, fostering shared decision-making and providing a clearer framework for assessing cancer risk.

### External validation

Furthermore, an external validation using data from a recently established RDC at QEH in southeast London showed that cancer patients at QEH were mostly White and tended to have lower socioeconomic status compared with those at Guy’s RDC. The validation also confirmed both scores predicted increased odds of cancer when analysed continuously (OR 1.16 (95% CI 1.06 to 1.27) and OR 1.28 (95% CI 1.15 to 1.42) for LCA and LASSO scores, respectively). However, when the score variable was dichotomised, the association for the LCA score was lost. This difference could be attributed to the broad range of non-cancer diagnoses within our patient population. These findings underscore the importance of considering the context and acknowledging the potential variations across different clinical settings.

### Strengths and limitations

Strengths of our study include our large study population. We collected thorough and reliable clinical and sociodemographic data, enhancing the robustness of our analysis. Specifically, our prospective data is detailed and of high quality. Moreover, the simplicity of the blood tests used allows for easy replication in primary care settings to aid in referral decisions. Furthermore, our study examines NSS comprehensively, not limited to weight loss alone, as seen in most previous publications. Nevertheless, our study has limitations. First, SKLC, SLLC, SFLC, ferritin and ESR exhibited over 40% missing data, a threshold beyond which caution is warranted when employing imputation techniques, as suggested by prior research.^24 25^ Second, LCA can only be used with observations (ie, patients) with complete predictive variables (ie, biomarkers), potentially introducing selection bias. However, comparisons between our complete case dataset used for LCA and our full dataset had comparable clinical and sociodemographic characteristics, including blood test results, mitigating concerns for selection bias. Furthermore, the generalisability of the identified biomarker combinations may be constrained to our specific patient population due to variations in RDC models. For instance, RDCs focusing on gastrointestinal conditions may encompass different patient demographics and disease profiles. Additionally, biomarkers were handled differently in the two models (continuous in LASSO, binary in LCA), which may limit direct comparability of the resulting scores. Another limitation is that the scores use clinical cut-offs, which can result in inflated scores for patients with only slightly elevated biomarkers. This may not accurately reflect their clinical significance.

Future research should involve validating both LCA and LASSO in external datasets with diverse clinical models, such as GI-led services. Replicating these analyses and their variable selection methods in varied settings will help assess the biomarkers’ generalisability and may identify additional biomarkers relevant to other clinical contexts.

Furthermore, all biomarkers identified, including SFLC, should be considered to be made accessible not only to other NSS services but also to primary care clinicians. Currently, SFLC tests are often restricted to haematology specialists due to their association with conditions like multiple myeloma. However, given their potential role as inflammation markers, their use could be expanded to other NSS services or even primary care settings. Nevertheless, further validation studies are necessary to support these suggestions, considering the cost implications associated with these tests.

Moreover, this study assessed the LCA and LASSO risk scores within the RDC setting. However, these scores could also be highly valuable in primary care, where they may aid clinicians in identifying patients at higher risk of cancer earlier. By flagging these high-risk patients, primary care clinicians could facilitate timely referrals to NSS services, optimising the diagnostic pathway and potentially improving patient outcomes. In the long term, integrating these risk scores into primary care could enhance patient management by helping identify high-risk patients before referral. For those assessed as low risk, the scores may support conservative monitoring strategies, thereby reducing unnecessary referrals and alleviating healthcare burdens. Before broader implementation, however, it is essential to validate these risk scores within primary care settings. This validation process will confirm whether the scores perform effectively in this context; if necessary, adjustments such as the addition or removal of specific biomarkers could improve their relevance and accuracy. Such steps will ensure that the scores reliably identify high-risk patients while minimising unwarranted referrals.

## Conclusion

In conclusion, our study demonstrates that our biomarker scores effectively identify patients at higher risk of cancer. Both scores emphasised the importance of inflammatory markers such as CRP, ESR and SKLC. LCA included a broader range of biomarkers (eg, WCC, Hb, NLR, neutrophils, albumin, SLLC), leading to higher sensitivity, while LASSO focused on fewer markers (eg, platelets, ALP, LDH) and achieved higher specificity. In addition, while our models were primarily designed for cancer detection, the biomarkers included are indicative of other serious benign conditions commonly encountered in our patient population. This suggests that our biomarker sets could also prove useful in detecting patients with severe benign conditions, enhancing the comprehensive management of individuals presenting with NSS in the RDC setting. However, it is imperative to interpret these findings cautiously and in conjunction with other clinical characteristics. By recognising the significance of these biomarkers, clinicians can enhance early detection and intervention strategies, ultimately improving patient outcomes with NSS.

## Supplementary material

10.1136/bmjopen-2025-099967 online supplemental file 1

## Data Availability

Data are available upon reasonable request.
