# Supplementary material for: Exploring a panel of serum biomarkers for cancer risk in patients with non-specific symptoms: a comparative analysis of feature selection methods
Source: BMJ Open. 2025 Dec 10;15(12):e099967. doi: 10.1136/bmjopen-2025-099967 (PMC12699618; doi:10.1136/bmjopen-2025-099967)

Supplementary Table 1. Summary of Serum Biomarker Measurements

| **Biomarker** | **Sample type** | **Analytical method / platform** | **Units** | **Limit of detection (LOD)*** |
| --- | --- | --- | --- | --- |
| White cell count (WCC) | Whole blood (EDTA) | Automated haematology analyser | ×10⁹/L | N/A |
| Haemoglobin (Hb) | Whole blood (EDTA) | Automated haematology analyser | g/L | N/A |
| Platelets | Whole blood (EDTA) | Automated haematology analyser | ×10⁹/L | N/A |
| ALP | Serum | Automated clinical chemistry | IU/L | <5 |
| ALT | Serum | Automated clinical chemistry | IU/L | <5 |
| Albumin | Serum | Automated clinical chemistry | g/L | <2 |
| Bilirubin | Serum | Automated clinical chemistry | µmol/L | <1 |
| Creatinine | Serum | Automated clinical chemistry | µmol/L | <10 |
| Calcium | Serum | Automated clinical chemistry | mmol/L | <0.1 |
| LDH | Serum | Automated clinical chemistry | IU/L | <10 |
| CRP | Serum | Immunoturbidimetry | mg/L | 0.1 |
| Ferritin | Serum | Immunoassay | µg/L | 5 |
| ESR | Whole blood (citrate) | Westergren method | mm/hr | N/A |
| SKLC / SLLC | Serum | Immunoassay | mg/L | 1 |
| PSA | Serum | Immunoassay | µg/L | 0.01 |
| CA125 | Serum | Immunoassay | U/mL | 1 |
| eGFR | Calculated from creatinine | CKD-EPI formula | mL/min/1.73m² | N/A |

*LOD = approximate lower limit of detection.

Supplementary Table 2. Clinical cut-off categories of biomarkers included in latent class analysis.

| **Biomarker** | **Clinical cut-offs** |
| --- | --- |
| *White cell count (WCC)* | - Low: < 4 - Normal: 4-11 - High: >11 |
| *Haemoglobin (Hb)* | - Low: <120 - Normal: 120-150 - High: >150 |
| *Platelets* | - Low: <150 - Normal 150-450 - High: >450 |
| *Neutrophils* | - Low: <1.5 - Normal: 1.5-7 - High: >7 |
| *Lymphocytes* | - Low: 1.2 - Normal: 1.2-3.5 - High: >3.5 |
| *Calcium* | - Low: <2.15 - Normal: 2.15-2.55 - High: >2.55 |
| *Bilirubin* | - Normal: - High: |
| *Alkaline phosphatase (ALP)* | - Low: <35 - Normal: 35-129 - High: >129 |
| *Alanine transaminase (ALT)* | - Low: <4 - Normal: 4-40 - High: >40 |
| *Albumin* | - Low: <40 - Normal: 40-52 - High: >52 |
| *Creatinine* | - Low: <45 - Normal: 45-85 - High: >85 |
| *Lactate dehydrogenase (LDH)* | - Low: <135 - Normal: 135-214 - High: >214 |
| *C-reactive protein (CRP)* | - Normal: <4 - High: >4 |
| *Ferritin* | - Low: <22 - Normal: 22-275 - High: >275 |
| *Erythrocyte sedimentation rate (ESR)* | - Normal: <15 - High: >15 |
| *Serum Kappa Light Chains (SKLC)* | - Low: <3.3 - Normal: 3.3-19.4 - High: >19.4 |
| *Serum Lambda Light Chains (SLLC)* | - Low: 5.7 - Normal: 5.7-26.3 - High: >26.3 |
| *Serum Free Light Chain Ratio (SFLC)* | - Low: <0.26 - Normal: 0.26-1.65 - High: >1.65 |
| *Neutrophil to lymphocyte ratio (NLR)* | - Normal: <4 - High: >4 |

Supplementary Table 3. Missing data ranges for biomarkers included in variable selection analyses.

| **Biomarker** | **% missing** |
| --- | --- |
| Albumin | 17.5 |
| Alkaline Phosphatase | 17.6 |
| Alanine aminotransferase | 18.3 |
| Bilirubin | 18.1 |
| C-reactive protein | 22.0 |
| Corrected calcium | 20.6 |
| Creatinine | 16.0 |
| Erythrocyte sedimentation rate | 47.0 |
| Ferritin | 49.0 |
| Haemoglobin | 9.0 |
| Lactate dehydrogenase | 38.0 |
| Neutrophil to lymphocyte ratio | 16.0 |
| Neutrophils | 15.5 |
| Platelets | 9.0 |
| Serum Kappa Light Chain | 57.0 |
| Serum Lamba Light Chain | 57.0 |
| Serum Light Chain Ratio | 57.0 |
| White Cell Count | 13.9 |

Supplementary Table 4. TRIPOD Checklist: Prediction Model Development and Validation.

| **Section/Topic** | **Item** |  | **Checklist Item** | **Page** |
| --- | --- | --- | --- | --- |
| **Title and abstract** | | | | |
| Title | 1 | D;V | Identify the study as developing and/or validating a multivariable prediction model, the target population, and the outcome to be predicted. | 1 |
| Abstract | 2 | D;V | Provide a summary of objectives, study design, setting, participants, sample size, predictors, outcome, statistical analysis, results, and conclusions. | 2 |
| **Introduction** | | | | |
| Background and objectives | 3a | D;V | Explain the medical context (including whether diagnostic or prognostic) and rationale for developing or validating the multivariable prediction model, including references to existing models. | 4, 5 |
|  | 3b | D;V | Specify the objectives, including whether the study describes the development or validation of the model or both. | 4, 5 |
| **Methods** | | | | |
| Source of data | 4a | D;V | Describe the study design or source of data (e.g., randomized trial, cohort, or registry data), separately for the development and validation data sets, if applicable. | 5-8 |
|  | 4b | D;V | Specify the key study dates, including start of accrual; end of accrual; and, if applicable, end of follow-up. | 5-8 |
| Participants | 5a | D;V | Specify key elements of the study setting (e.g., primary care, secondary care, general population) including number and location of centres. | 5-8 |
|  | 5b | D;V | Describe eligibility criteria for participants. | NA |
|  | 5c | D;V | Give details of treatments received, if relevant. | NA |
| Outcome | 6a | D;V | Clearly define the outcome that is predicted by the prediction model, including how and when assessed. | 5, 6 |
|  | 6b | D;V | Report any actions to blind assessment of the outcome to be predicted. | NA |
| Predictors | 7a | D;V | Clearly define all predictors used in developing or validating the multivariable prediction model, including how and when they were measured. | 5-8 |
|  | 7b | D;V | Report any actions to blind assessment of predictors for the outcome and other predictors. | NA |
| Sample size | 8 | D;V | Explain how the study size was arrived at. | 5 |
| Missing data | 9 | D;V | Describe how missing data were handled (e.g., complete-case analysis, single imputation, multiple imputation) with details of any imputation method. | 6-8 |
| Statistical analysis methods | 10a | D | Describe how predictors were handled in the analyses. | 5-8 |
|  | 10b | D | Specify type of model, all model-building procedures (including any predictor selection), and method for internal validation. | 5-8 |
|  | 10c | V | For validation, describe how the predictions were calculated. | 7-8 |
|  | 10d | D;V | Specify all measures used to assess model performance and, if relevant, to compare multiple models. | 7-8 |
|  | 10e | V | Describe any model updating (e.g., recalibration) arising from the validation, if done. | NA |
| Risk groups | 11 | D;V | Provide details on how risk groups were created, if done. | NA |
| Development vs. validation | 12 | V | For validation, identify any differences from the development data in setting, eligibility criteria, outcome, and predictors. | NA |
| **Results** | | | | |
| Participants | 13a | D;V | Describe the flow of participants through the study, including the number of participants with and without the outcome and, if applicable, a summary of the follow-up time. A diagram may be helpful. | 9 |
|  | 13b | D;V | Describe the characteristics of the participants (basic demographics, clinical features, available predictors), including the number of participants with missing data for predictors and outcome. | 9 |
|  | 13c | V | For validation, show a comparison with the development data of the distribution of important variables (demographics, predictors and outcome). | 11 |
| Model development | 14a | D | Specify the number of participants and outcome events in each analysis. | 9-11 |
|  | 14b | D | If done, report the unadjusted association between each candidate predictor and outcome. | 9-11 |
| Model specification | 15a | D | Present the full prediction model to allow predictions for individuals (i.e., all regression coefficients, and model intercept or baseline survival at a given time point). | 9-11 |
|  | 15b | D | Explain how to the use the prediction model. | 9-11 |
| Model performance | 16 | D;V | Report performance measures (with CIs) for the prediction model. | 9-11 |
| Model-updating | 17 | V | If done, report the results from any model updating (i.e., model specification, model performance). | NA |
| **Discussion** | | | | |
| Limitations | 18 | D;V | Discuss any limitations of the study (such as nonrepresentative sample, few events per predictor, missing data). | 14 |
| Interpretation | 19a | V | For validation, discuss the results with reference to performance in the development data, and any other validation data. | 12-15 |
|  | 19b | D;V | Give an overall interpretation of the results, considering objectives, limitations, results from similar studies, and other relevant evidence. | 12-16 |
| Implications | 20 | D;V | Discuss the potential clinical use of the model and implications for future research. | 12-16 |
| **Other information** | | | | |
| Supplementary information | 21 | D;V | Provide information about the availability of supplementary resources, such as study protocol, Web calculator, and data sets. | 1-15 |
| Funding | 22 | D;V | Give the source of funding and the role of the funders for the present study. | 16 |

Supplementary Table 5. Patient sociodemographic and biomarker characteristics by cancer status.

|  | **Cancer**  **n=364**  **6.3%**  **n (%)** | **No Cancer**  **n=5,457**  **93.7%**  **n (%)** | **P-value** | **Total**  **n=5,821**  **n (%)** |
| --- | --- | --- | --- | --- |
| ***Sex*** | | | | |
| ***Male*** | 201 (55.2) | 2,206 (40.4) | <0.001 | 2,407 (41.4) |
| ***Female*** | 163 (44.8) | 3,251 (59.6) |  | 3,414 (58.6) |
| ***Age*** | | | | |
| ***Mean (SD)*** | 68 (13) | 61 (15) | <0.001 | 62 (15) |
| ***18-30*** | 6 (1.7) | 197 (3.6) | <0.001 | 203 (3.5) |
| ***30-50*** | 21 (5.8) | 1,055 (19.3) |  | 1,076 (18.5) |
| ***50-70*** | 168 (46.1) | 2,396 (43.9) |  | 2,564 (44) |
| ***>70*** | 169 (46.4) | 1,809 (33.2) |  | 1,978 (34) |
| ***Ethnicity*** | | | | |
| ***White*** | 208 (43.1) | 2,299 (42.1) | <0.001 | 2,507 (43.1) |
| ***Black*** | 44 (12.1) | 1,018 (18.7) |  | 1,062 (18.2) |
| ***Asian*** | 9 (2.5) | 316 (5.8) |  | 325 (5.6) |
| ***Other*** | 13 (3.6) | 215 (3.9) |  | 228 (3.9) |
| ***Not known*** | 90 (24.7) | 1609 (29.5) |  | 1,699 (29.2) |
| ***Index of Multiple Deprivation*** | | | | |
| ***Low (<4)*** | 136 (37.3) | 2,261 (41.4) | <0.001 | 2,397 (41.2) |
| ***Middle (4-7)*** | 170 (46.7) | 2,447 (44.8) |  | 2,617 (45) |
| ***High (>7)*** | 52 (14.2) | 723 (13.3) |  | 775 (13.3) |
| ***Not known*** | 6 (1.6) | 26 (0.5) |  | 32 (0.5) |
| ***White cell count*** | | | | |
| ***Mean (SD)*** | 7.5 (2.9) | 6.8 (4.7) | 0.003 | 6.8 (4.6) |
| ***Low*** | 20 (5.5) | 435 (8) | <0.001 | 455 (7.8) |
| ***Normal*** | 294 (80.8) | 3,653 (66.9) |  | 3,947 (67.8) |
| ***High*** | 33 (9.1) | 214 (3.9) |  | 247 (4.2) |
| ***Missing*** | 17 (4.7) | 1,155 (21.2) |  | 1,172 (20.1) |
| ***Haemoglobin*** | | | | |
| ***Mean (SD)*** | 124.6 (19.7) | 129.6 (16.7) | <0.001 | 129.2 (17) |
| ***Low*** | 137 (37.6) | 1,152 (21.1) | <0.001 | 1,289 (22.1) |
| ***Normal*** | 186 (51.1) | 2,764 (50.7) |  | 2,950 (50.7) |
| ***High*** | 27 (7.4) | 404 (7.4) |  | 431 (7.4) |
| ***Missing*** | 14 (3.9) | 1,137 (20.8) |  | 14 (3.9) |
| ***Platelets*** | | | | |
| ***Mean (SD)*** | 302.2 (156.3) | 266.8 (95.6) | <0.001 | 269.4 (101.8) |
| ***Low*** | 27 (7.4) | 225 (4.1) | <0.001 | 252 (4.3) |
| ***Normal*** | 279 (76.7) | 3,858 (70.7) |  | 4,137 (71.1) |
| ***High*** | 42 (11.5) | 200 (3.7) |  | 242 (4.2) |
| ***Missing*** | 16 (4.4) | 1,174 (21.5) |  | 1,190 (20.4) |
| ***Neutrophils*** | | | | |
| ***Mean (SD)*** | 5.1 (2.5) | 4.4 (5.9) | 0.024 | 4.4 (5.7) |
| ***Low*** | 6 (1.6) | 135 (2.5) | <0.001 | 141 (2.4) |
| ***Normal*** | 281 (77.2) | 3,731 (68.4) |  | 4,012 (68.9) |
| ***High*** | 59 (16.2) | 359 (6.6) |  | 418 (7.2) |
| ***Missing*** | 18 (5) | 1,232 (22.6) |  | 1,250 (21.5) |
| ***Lymphocytes*** | | | | |
| ***Mean (SD)*** | 1.6 (0.9) | 1.7 (1.6) | 0.053 | 1.7 (1.5) |
| ***Low*** | 128 (35.2) | 1,003 (18.4) | <0.001 | 1,131 (19.4) |
| ***Normal*** | 204 (56) | 3,120 (57.2) |  | 3,324 (57.1) |
| ***High*** | 9 (2.5) | 90 (1.7) |  | 99 (1.7) |
| ***Missing*** | 23 (6.3) | 1,244 (22.8) |  | 1,267 (21.8) |
| ***Neutrophil-to-lymphocyte ratio*** | | | | |
| ***Mean (SD)*** | 3.8 (2.6) | 2.9 (5.1) | 0.001 | 2.9 (4.9) |
| ***Normal*** | 223 (61.3) | 3,520 (64.5) | <0.001 | 3,743 (64.3) |
| ***High*** | 118 (32.4) | 678 (12.4) |  | 796 (13.7) |
| ***Missing*** | 23 (6.3) | 1,259 (23.1) |  | 1,282 (22) |
| ***Calcium*** | | | | |
| ***Mean (SD)*** | 2.5 (2.1) | 2.4 (3.8) | 0.874 | 2.4 (3.8) |
| ***Normal*** | 289 (79.3) | 3,648 (66.9) | <0.001 | 3,937 (67.6) |
| ***High*** | 39 (19.7) | 327 (6) |  | 366 (6.3) |
| ***Missing*** | 36 (9.9) | 1,482 (27.2) |  | 1,518 (26.1) |
| ***Bilirubin*** | | | | |
| ***Mean (SD)*** | 10.3 (16.7) | 8.9 (9) | 0.010 | 9 (9.8) |
| ***Normal*** | 316 (86.8) | 3,884 (71.2) | <0.001 | 4,200 (72.2) |
| ***High*** | 19 (5.2) | 164 (3) |  | 183 (3.1) |
| ***Missing*** | 29 (8) | 1,409 (25.8) |  | 1,438 (24.7) |
| ***Alkaline Phosphatase*** | | | | |
| ***Mean (SD)*** | 155.6 (261.1) | 86.9 (55.7) | <0.001 | 92.1 (91.2) |
| ***Normal*** | 234 (64.6) | 3,724 (68.2) | <0.001 | 3,959 (68) |
| ***High*** | 97 (26.7) | 348 (6.4) |  | 445 (7.7) |
| ***Missing*** | 32 (8.8) | 1,385 (25.4) |  | 1,417 (24.3) |
| ***Alanine Transaminase*** | | | | |
| ***Mean (SD)*** | 28.4 (41.1) | 23.2 (28.1) | 0.002 | 23.6 (29.4) |
| ***Normal*** | 293 (80.5) | 3,681 (67.4) | <0.001 | 3,974 (68.3) |
| ***High*** | 42 (11.5) | 334 (6.1) |  | 376 (6.5) |
| ***Missing*** | 29 (8) | 1,442 (26.4) |  | 1,471 (25.3) |
| ***Albumin*** | | | | |
| ***Mean (SD)*** | 42.1 (5.3) | 44.1 (4.7) | <0.001 | 44 (4.7) |
| ***Low*** | 114 (31.3) | 686 (12.6) | <0.001 | 800 (13.7) |
| ***Normal*** | 205 (56.3) | 3,249 (59.5) |  | 3,454 (59.3) |
| ***High*** | 4 (1.1) | 42 (0.8) |  | 46 (0.8) |
| ***Missing*** | 41 (11.3) | 1,480 (27.1) |  | 1,521 (26.1) |
| ***Creatinine*** | | | | |
| ***Mean (SD)*** | 82.2 (29.2) | 77.9 (29.9) | 0.011 | 78.3 (29.9) |
| ***Low*** | 12 (3.3) | 115 (2.1) | <0.001 | 127 (2.2) |
| ***Normal*** | 209 (57.4) | 2,861 (52.4) |  | 3,070 (52.7) |
| ***High*** | 119 (32.7) | 1,170 (21.5) |  | 1,289 (22.1) |
| ***Missing*** | 24 (6.6) | 1,311 (24) |  | 1,335 (22.9) |
| ***Estimated glomerular filtration rate*** | | | | |
| ***Mean (SD)*** | 79.7 (26.7) | 81.4 (24.5) | 0.182 | 81.3 (24.7) |
| ***Low*** | 128 (35.2) | 1,349 (24.7) | <0.001 | 1,477 (25.4) |
| ***Normal*** | 212 (58.2) | 2,786 (51.1) |  | 2,998 (51.5) |
| ***Missing*** | 24 (6.6) | 1,322 (24.2) |  | 1,346 (23.1) |
| ***Lactate dehydrogenase*** | | | | |
| ***Mean (SD)*** | 285.9 (321.7) | 198.3 (76.1) | <0.001 | 204.8 (116.1) |
| ***Normal*** | 142 (39) | 2,356 (43.2) | <0.001 | 2,498 (43) |
| ***High*** | 120 (33) | 942 (17.3) |  | 1,062 (18.2) |
| ***Missing*** | 102 (28) | 2,159 (39.6) |  | 2,261 (38.8) |
| ***C-Reactive protein*** | | | | |
| ***Mean (SD)*** | 26.9 (47.1) | 8.1 (20.6) | <0.001 | 9.5 (24.2) |
| ***Normal*** | 131 (36) | 2,718 (49.8) | <0.001 | 2,849 (49) |
| ***High*** | 185 (50.8) | 1,127 (20.7) |  | 1,312 (22.5) |
| ***Missing*** | 48 (13.2) | 1,612 (29.5) |  | 1,660 (28.5) |
| ***Ferritin*** | | | | |
| ***Mean (SD)*** | 718 (6356) | 169 (310) | <0.001 | 209 (1742) |
| ***Normal*** | 146 (40.6) | 2,346 (43) | <0.001 | 2,492 (42.8) |
| ***High*** | 71 (19.5) | 421 (7.7) |  | 492 (8.5) |
| ***Missing*** | 147 (40.4) | 2,690 (49.3) |  | 2,837 (48.7) |
| ***Erythrocyte sedimentation rate*** | | | | |
| ***Mean (SD)*** | 32.1 (31.8) | 19.5 (24.7) | <0.001 | 20.4 (25.4) |
| ***Normal*** | 95 (26.1) | 1,774 (32.5) | <0.001 | 1,869 (32.1) |
| ***High*** | 134 (36.8) | 1,162 (21.3) |  | 1,296 (22.3) |
| ***Missing*** | 135 (37.1) | 2,521 (46.2) |  | 2,656 (45.6) |
| ***Serum Kappa Light Chains*** | | | | |
| ***Mean (SD)*** | 76.2 (230) | 28 (26.5) | <0.001 | 31.3 (66.7) |
| ***Normal*** | 53 (14.3) | 1,070 (19.6) | <0.001 | 1,122 (19.3) |
| ***High*** | 134 (36.8) | 1,239 (26.4) |  | 1,573 (27) |
| ***Missing*** | 177 (48.6) | 2,948 (54) |  | 3,125 (53.7) |
| ***Serum Lambda Light Chains*** | | | | |
| ***Mean (SD)*** | 172.8 (1818) | 38.3 (821.1) | 0.054 | 47.6 (925.9) |
| ***Normal*** | 114 (30.2) | 2,042 (37.2) | <0.001 | 2,156 (37) |
| ***High*** | 73 (20.1) | 460 (8.4) |  | 533 (9.2) |
| ***Missing*** | 177 (48.6) | 2,955 (54.2) |  | 3,132 (53.8) |
| ***Serum Free Light Chain Ratio*** | | | | |
| ***Mean (SD)*** | 16.4 (90) | 1.4 (2.2) | <0.001 | 2.5 (24.1) |
| ***Normal*** | 140 (36.3) | 2,092 (38.3) | <0.001 | 2,232 (38.3) |
| ***High*** | 50 (13.7) | 433 (7.9) |  | 483 (8.3) |
| ***Missing*** | 174 (47.8) | 2,932 (53.7) |  | 3,106 (53.4) |

*SD, standard deviation.*

Supplementary Table 6. Sociodemographic and biomarker characteristics by cancer status within the LCA subset.

|  | | **Cancer**  **n=102**  **6.6%**  **n (%)** | **No Cancer**  **n=1,443**  **93.4%**  **n (%)** | **P-value** | **Total**  **n=1,545**  **n (%)** |
| --- | --- | --- | --- | --- | --- |
| ***Sex*** | | | | | |
| ***Male*** | | 54 (53) | 591 (40.9) | 0.018 | 645 (41.7) |
| ***Female*** | | 48 (47) | 852 (59.1) |  | 900 (58.3) |
| ***Age*** | | | | | |
| ***Mean (SD)*** | | 68 (12) | 61 (15) | <0.001 | 62 (15) |
| ***18-30*** | | 1 (1) | 49 (3.4) | 0.001 | 50 (3.2) |
| ***30-50*** | | 6 (5.9) | 257 (17.8) |  | 263 (17) |
| ***50-70*** | | 45 (44.1) | 678 (47) |  | 723 (46.8) |
| ***>70*** | | 50 (49) | 459 (31.8) |  | 509 (33) |
| ***Ethnicity*** | | | | | |
| ***White*** | | 56 (54.9) | 723 (50.1) | 0.003 | 779 (50.4) |
| ***Black*** | | 16 (15.7) | 309 (21.4) |  | 325 (21) |
| ***Asian*** | | 1 (1) | 107 (7.4) |  | 108 (7) |
| ***Other*** | | 3 (2.9) | 88 (6.1) |  | 91 (5.9) |
| ***Not known*** | | 26 (25.5) | 216 (15) |  | 242 (15.6) |
| ***Index of Multiple Deprivation*** | | | | | |
| ***Low (<4)*** | | 41 (40.2) | 620 (43) | 0.001 | 661 (42.8) |
| ***Middle (4-7)*** | | 48 (47.1) | 577 40) |  | 625 (40.4) |
| ***High (>7)*** | | 11 (10.8) | 236 (16.4) |  | 247 (16.0) |
| ***Not known*** | | 2 (1.9) | 10 (0.6) |  | 12 (0.8) |
| ***White cell count*** | | | | | |
| ***Mean (SD)*** | | 7.5 (3.3) | 6.8 (9.5) | 0.402 | 6.8 (3.1) |
| ***Low*** | | 10 (9.8) | 137 (9.5) | 0.022 | 147 (9.5) |
| ***Normal*** | | 81 (79.4) | 1,239 (85.9) |  | 1,320 (85.4) |
| ***High*** | | 11 (10.8) | 67 (4.6) |  | 78 (5.1) |
| ***Haemoglobin*** | | | | | |
| ***Mean (SD)*** | | 122.3 (19.1) | 129.5 (16.8) | <0.001 | 129 (17) |
| ***Low*** | | 44 (43.1) | 399 (27.7) | 0.003 | 443 (28.7) |
| ***Normal*** | | 53 (52) | 917 (63.6) |  | 970 (62.8) |
| ***High*** | | 5 (4.9) | 127 (8.8) |  | 132 (8.5) |
| ***Platelets*** | | | | | |
| ***Mean (SD)*** | | 287 (146) | 273 (102) | 0.191 | 274 (105) |
| ***Low*** | | 11 (10.8) | 73 (5) | <0.001 | 84 (5.4) |
| ***Normal*** | | 77 (75.5) | 1,287 (89.2) |  | 1,364 (88.3) |
| ***High*** | | 14 (13.7) | 83 (5.8) |  | 97 (6.3) |
| ***Neutrophils*** | | | | | |
| ***Mean (SD)*** | | 4.9 (2.7) | 4.4 (7.1) | 0.511 | 4.4 (6.9) |
| ***Low*** | | 3 (2.9) | 41 (2.8) | 0.142 | 44 (2.8) |
| ***Normal*** | | 85 (83.3) | 1,285 (89.1) |  | 1,370 (88.7) |
| ***High*** | | 14 (13.8) | 117 (8.1) |  | 131 (8.5) |
| ***Lymphocytes*** | | | | | |
| ***Mean (SD)*** | | 1.6 (1.1) | 1.8 (2.1) | 0.535 | 1.8 (2) |
| ***Low*** | | 41 (40.2) | 354 (24.5) | <0.001 | 395 (25.6) |
| ***Normal*** | | 56 (54.9) | 1,060 (73.5) |  | 1,116 (72.2) |
| ***High*** | | 5 (4.9) | 29 (2) |  | 34 (2.2) |
| ***Neutrophil to lymphocyte ratio*** | | | | | |
| ***Mean (SD)*** | | 3.7 (3) | 3 (6.1) | 0.208 | 3 (5.9) |
| ***Normal*** | | 71 (69.6) | 1,201 (83.2) | <0.001 | 1,272 (82.3) |
| ***High*** | | 31 (30.4) | 242 (16.8) |  | 273 (17.7) |
| ***Calcium*** | | | | | |
| ***Mean (SD)*** | | 2.7 (3.8) | 2.5 (6.2) | 0.766 | 2.5 (6.1) |
| ***Normal*** | | 92 (90.2) | 1,315 (91.1) | 0.002 | 1.407 (91.1) |
| ***High*** | | 10 (9.8) | 128 (8.9) |  | 138 (8.9) |
| ***Bilirubin*** | | | | | |
| ***Mean (SD)*** | | 9.5 (6) | 9.4 (11.1) | 0.958 | 9.4 (10.9) |
| ***Normal*** | | 97 (95.1) | 1,379 (95.5) | 0.852 | 1,476 (95.5) |
| ***High*** | | 5 (4.9) | 64 (4.5) |  | 69 (5.5) |
| ***Alkaline Phosphatase*** | | | | | |
| ***Mean (SD)*** | | 143 (154) | 89 (54) | <0.001 | 92.6 (66.9) |
| ***Normal*** | | 71 (69.6) | 1,318 (91.3) | <0.001 | 1,389 (89.9) |
| ***High*** | | 31 (30.4) | 125 (8.7) |  | 156 (10.1) |
| ***Alanine Transaminase*** | | | | | |
| ***Mean (SD)*** | | 25.6 (32.4) | 23.7 (27) | 0.921 | 23.8 (27.4) |
| ***Normal*** | | 93 (91.2) | 1,313 (91) | 0.485 | 1,406 (91) |
| ***High*** | | 9 (8.8) | 130 (9) |  | 139 (9) |
| ***Albumin*** | | | | | |
| ***Mean (SD)*** | | 42.3 (5.7) | 43.5 (4.8) | 0.020 | 23.8 (27.4) |
| ***Low*** | | 30 (29.4) | 329 (22.8) | 0.293 | 359 (23.2) |
| ***Normal*** | | 66 (64.7) | 1,092 (75.7) |  | 1,162 (75.2) |
| ***High*** | | 2 (2) | 22 (1.5) |  | 24 (1.6) |
| ***Creatinine*** | | | | | |
| ***Mean (SD)*** | | 82.2 (30) | 76.6 (25.4) | 0.031 | 76.9 (25.7) |
| ***Low*** | | 5 (4.9) | 39 (2.7) | 0.051 | 44 (2.9) |
| ***Normal*** | | 61 (59.8) | 1,022 (70.8) |  | 1,083 (70) |
| ***High*** | | 36 (35.3) | 382 (26.5) |  | 418 (27.1) |
| ***Lactate dehydrogenase*** | | | | | |
| ***Mean (SD)*** | | 297.7 (301.4) | 197.6 (58.3) | <0.001 | 204.2 (98.7) |
| ***Normal*** | | 58 (56.9) | 1,035 (71.7) | <0.001 | 1,093 (70.7) |
| ***High*** | | 44 (43.1) | 408 (28.3) |  | 452 (29.3) |
| ***C-Reactive protein*** | | | | | |
| ***Mean (SD)*** | | 25.6 (44.5) | 7.9 (19.4) | <0.001 | 9.1 (22.4) |
| ***Normal*** | | 54 (52.9) | 1,033 (71.6) | <0.001 | 1,087 (70.4) |
| ***High*** | | 48 (47.1) | 410 (28.4) |  | 458 (29.6) |
| ***Ferritin*** | | | | | |
| ***Mean (SD)*** | | 281.6 (350.1) | 169.5 (322.8) | <0.001 | 176.9 (325.7) |
| ***Normal*** | | 69 (67.6) | 1,220 (84.5) | <0.001 | 1,289 (83.4) |
| ***High*** | | 33 (32.4) | 223 (15.5) |  | 256 (16.6) |
| ***Erythrocyte sedimentation rate*** | | | | | |
| ***Mean (SD)*** | | 33.3 (33.8) | 18.9 (24.5) | <0.001 | 19.9 (25.4) |
| ***Normal*** | | 43 (42.2) | 891 (61.8) | <0.001 | 934 (60.5) |
| ***High*** | | 59 (57.8) | 552 (38.3) |  | 611 (39.5) |
|  | ***Serum Kappa Light Chains*** | | | | |
| ***Mean (SD)*** | | 46.9 (72.3) | 27.7 (20.7) | <0.001 | 29 (27.7) |
| ***Normal*** | | 30 (29.4) | 578 (40) | 0.102 | 608 (39.3) |
| ***High*** | | 72 (70.6) | 865 (60) |  | 937 (60.7) |
| ***Serum Lambda Light Chains*** | | | | | |
| ***Mean (SD)*** | | 272.7 (2459) | 22.4 (43.1) | <0.001 | 38.9 (633) |
| ***Normal*** | | 63 (61.8) | 1,162 (80.5) | <0.001 | 1,225 (79.3) |
| ***High*** | | 39 (38.2) | 281 (19.5) |  | 320 (20.7) |
| ***Serum Free Light Chain Ratio*** | | | | | |
| ***Mean (SD)*** | | 4 (18.1) | 1.4 (2.4) | <0.001 | 1.5 (5.2) |
| ***Normal*** | | 77 (75) | 1,193 (82,7) | <0.001 | 1,270 (82.2) |
| ***High*** | | 25 (25) | 250 (17.3) |  | 275 (17.8) |

SD, standard deviation.

Supplementary Table 7. Logistic regression for risk of cancer among LCA-derived classes.

|  | **Odds Ratios (95%CI)** |
| --- | --- |
| **Class 1 (“Normal”)** | 1.00 (Ref) |
| **Class 2 (“Serious condition”)** | 2.81 (1.78-4.45) |
| **Class 3 (“Very serious condition”)** | 4.26 (2.26-8.02) |

Supplementary Table 8. Distribution of final diagnosis across latent classes.

|  | **Class 1 (“Normal”)**  **n=868**  **n (%)** | **Class 2 (“Serious condition”)**  **n=456**  **n (%)** | **Class 3 (“Very serious condition”)**  **n=107**  **n (%)** |
| --- | --- | --- | --- |
| **Cancer** | 34 (4) | 47 (10.3) | 16 (15.0) |
| **Serious benign condition** | 245 (28.2) | 187 (41.0) | 44 (41.1) |
| **Non-serious benign condition** | 356 (41) | 134 (29.4) | 34 (31.8) |
| **None** | 223 (26.8) | 88 (19.3) | 13 (12.1) |

Supplementary Table 9. Logistic Regression Results for Scores Analysed as Continuous Variables (Per 1 SD Increase).

| **Score** | **Unadjusted** | **Adjusted*** | **External validation** | **External validation adjusted*** |
| --- | --- | --- | --- | --- |
|  | **OR (95%C)** | **OR (95%C))** | **OR (95%C)** | **OR (95%C)** |
| **Latent Class Analysis Scores** | | | | |
| **Continuous** | 1.80 (1.65-1.96) | 1.71 (1.57-1.87) | 1.67 (1.27-2.17) | 1.56 (1.18-2.05) |
| **LASSO Scores** | | | | |
| **Continuous** | 1.98 (1.81-2.16) | 1.93 (1.76-2.11) | 2.06 (1.57-2.72) | 1.98 (1.49-2.63) |

Supplementary Table 10. Net Benefit (Decision Curve Analysis).

|  | Net Benefit | | | |
| --- | --- | --- | --- | --- |
| Decision Threshold | Treat All | Treat None | LCA | LASSO |
| 1% | 0.053 | 0.000 | 0.053 | 0.053 |
| 2% | 0.043 | 0.000 | 0.043 | 0.043 |
| 3% | 0.034 | 0.000 | 0.036 | 0.036 |
| 4% | 0.023 | 0.000 | 0.031 | 0.031 |
| 5% | 0.013 | 0.000 | 0.027 | 0.026 |
| 6% | 0.003 | 0.000 | 0.022 | 0.022 |
| 7% | -0.008 | 0.000 | 0.018 | 0.018 |
| 8% | -0.019 | 0.000 | 0.014 | 0.013 |
| 9% | -0.030 | 0.000 | 0.011 | 0.010 |
| 10% | -0.042 | 0.000 | 0.009 | 0.008 |
| 11% | -0.053 | 0.000 | 0.006 | 0.006 |
| 12% | -0.065 | 0.000 | 0.005 | 0.004 |
| 13% | -0.078 | 0.000 | 0.003 | 0.004 |
| 14% | -0.090 | 0.000 | 0.001 | 0.001 |
| 15% | -0.103 | 0.000 | -0.001 | 0.000 |

LCA, latent class analysis; LASSO, Least Absolute Shrinkage and Selection Operator.

Supplementary Table 11. Patient characteristics by cancer status (QEH).

|  | **No Cancer**  **n=535**  **93.4%**  **n (%)** | **Cancer**  **n=38**  **6.6%**  **n (%)** | **Total**  **n= 573**  **n (%)** |
| --- | --- | --- | --- |
| ***Sex*** |  |  |  |
| ***Male*** | 226 (42.2) | 16 (42.1) | 242 (42.2) |
| ***Female*** | 309 (57.8) | 22 (57.9) | 331 (57.8) |
| ***Age*** |  |  |  |
| ***Mean (SD)*** | 63 (16) | 71 (13) | 63 (16) |
| ***18-30*** | 22 (4.1) | 0 | 22 (3.8) |
| ***30-50*** | 91 (17) | 3 (7.9) | 94 (16.4) |
| ***50-70*** | 218 (40.8) | 14 (36.8) | 232 (40.5) |
| ***>70*** | 204 (38.1) | 21 (55.3) | 225 (39.3) |
| ***Ethnicity*** |  |  |  |
| ***White*** | 379 (70.8) | 33 (86.8) | 412 (71.9) |
| ***Black*** | 66 (12.3) | 2 (5.3) | 68 (11.9) |
| ***Asian*** | 53 (9.9) | 2 (5.3) | 55 (9.6) |
| ***Other*** | 37 (7) | 1 (2.6) | 37 (6.5) |
| ***IMD*** |  |  |  |
| ***Low (<4)*** | 178 (33.3) | 16 (42.1) | 194 (33.9) |
| ***Middle (4-7)*** | 276 (51.6) | 16 (42.1) | 292 (51) |
| ***High(>7)*** | 81 (15.1) | 6 (15.8) | 87 (15.1) |
| ***White cell count*** |  |  |  |
| ***Mean (SD)*** |  |  |  |
| ***Low*** | 22 (4.1) | 1 (2.6) | 23 (4) |
| ***Normal*** | 422 (78.9) | 31 (81.6) | 453 (79.1) |
| ***High*** | 55 (10.3) | 5 (13.2) | 60 (10.5) |
| ***Missing*** | 36 (6.7) | 1 (2.6) | 37 (6.5) |
| ***Haemoglobin*** |  |  |  |
| ***Mean (SD)*** |  |  |  |
| ***Low*** | 125 (23.4) | 18 (47.4) | 143 (25) |
| ***Normal*** | 377 (70.5) | 20 (52.6) | 397 (57.3) |
| ***Missing*** | 33 (6.2) | 0 | 33 (5.7) |
| ***Platelets*** |  |  |  |
| ***Mean (SD)*** |  |  |  |
| ***Low*** | 18 (3.4) | 2 (5.3) | 20 (3.5) |
| ***Normal*** | 432 (80.8) | 30 (79) | 462 (80.6) |
| ***High*** | 48 (9) | 6 (15.7) | 54 (9.4) |
| ***Missing*** | 37 (6.8) | 0 | 37 (6.5) |
| ***Neutrophils*** |  |  |  |
| ***Mean (SD)*** |  |  |  |
| ***Normal*** | 416 (77.8) | 29 (76.3) | 445 (77.7) |
| ***High*** | 77 (14.4) | 8 (21.1) | 85 (14.8) |
| ***Missing*** | 42 (7.9) | 1 (2.6) | 43 (7.5) |
| ***Lymphocytes*** |  |  |  |
| ***Mean (SD)*** |  |  |  |
| ***Low*** | 76 (14.2) | 9 (23.7) | 85 (14.8) |
| ***Normal*** | 393 (73.5) | 27 (71.1) | 420 (73.3) |
| ***High*** | 24 (4.5) | 0 | 24 (4.2) |
| ***Missing*** | 42 (7.8) | 2 (5.3) | 44 (7.7) |
| ***NLR*** |  |  |  |
| ***Mean (SD)*** |  |  |  |
| ***Normal*** | 414 (77.4) | 24 (63.2) | 438 (76.4) |
| ***High*** | 79 (14.8) | 12 (31.6) | 91 (15.9) |
| ***Missing*** | 42 (7.9) | 2 (5.3) | 44 (7.7) |
| ***Calcium*** |  |  |  |
| ***Mean (SD)*** |  |  |  |
| ***Normal*** | 462 (86.4) | 32 (84.2) | 494 (86.2) |
| ***High*** | 27 (5.1) | 4 (10.5) | 31 (5.4) |
| ***Missing*** | 46 (8.6) | 2 (5.3) | 48 (8.4) |
| ***Bilirubin*** |  |  |  |
| ***Mean (SD)*** |  |  |  |
| ***Normal*** | 467 (87.3) | 33 (86.8) | 500 (87.3) |
| ***High*** | 13 (2.4) | 2 (5.3) | 15 (2.6) |
| ***Missing*** | 55 (10.3) | 3 (7.9) | 58 (10.1) |
| ***Alkaline Phosphatase*** |  |  |  |
| ***Mean (SD)*** |  |  |  |
| ***Normal*** | 467 (87.3) | 33 (86.8) | 500 (87.3) |
| ***High*** | 13 (2.4) | 2 (5.3) | 15 (2.6) |
| ***Missing*** | 55 (10.3) | 3 (7.9) | 58 (10.1) |
| ***Alanine Transaminase*** |  |  |  |
| ***Mean (SD)*** |  |  |  |
| ***Normal*** | 342 (63.9) | 26 (68.4) | 368 (64.2) |
| ***High*** | 25 (4.7) | 0 | 25 (4.4) |
| ***Missing*** | 168 (31.4) | 12 (31.6) | 180 (31.4) |
| ***Albumin*** |  |  |  |
| ***Mean (SD)*** |  |  |  |
| ***Low*** | 70 (13.1) | 11 (29) | 81 (14.2) |
| ***Normal*** | 428 (80) | 25 (65.8) | 453 (79) |
| ***Missing*** | 37 (6.9) | 2 (5.2) | 39 (6.8) |
| ***eGFR*** |  |  |  |
| ***Mean (SD)*** |  |  |  |
| ***Low*** | 163 (30.5) | 14 (36.8) | 177 (30.9) |
| ***Normal*** | 325 (60.8) | 22 (57.9) | 347 (60.6) |
| ***Missing*** | 47 (8.8) | 2 (5.3) | 49 (8.5) |
| ***Lactate dehydrogenase*** |  |  |  |
| ***Mean (SD)*** |  |  |  |
| ***Normal*** | 230 (43) | 16 (42) | 246 (43) |
| ***High*** | 157 (29.4) | 12 (31.6) | 169 (29.5) |
| ***Missing*** | 148 (27.7) | 10 (26.4) | 158 (27.5) |
| ***C-Reactive protein*** |  |  |  |
| ***Mean (SD)*** |  |  |  |
| ***Normal*** | 351 (65.6) | 13 (34.2) | 364 (63.5) |
| ***High*** | 127 (23.7) | 21 (55.3) | 148 (25.8) |
| ***Missing*** | 57 (10.7) | 4 (10.5) | 61 (10.7) |
| ***Ferritin*** |  |  |  |
| ***Mean (SD)*** |  |  |  |
| ***Normal*** | 377 (76.5) | 19 (50) | 396 (69.1) |
| ***High*** | 88 (16.5) | 13 (34.2) | 101 (17.6) |
| ***Missing*** | 70 (13.1) | 6 (15.8) | 76 (13.3) |
| ***Erythrocyte sedimentation rate*** |  |  |  |
| ***Mean (SD)*** |  |  |  |
| ***Normal*** | 268 (50.1) | 10 (26.3) | 278 (48.5) |
| ***High*** | 217 (40.6) | 22 (57.9) | 239 (41.7) |
| ***Missing*** | 50 (9.4) | 6 (15.8) | 56 (9.8) |
| ***Serum Kappa Light Chains*** |  |  |  |
| ***Mean (SD)*** |  |  |  |
| ***Normal*** | 133 (24.8) | 9 (23.7) | 142 (24.8) |
| ***High*** | 271 (50.7) | 13 (34.2) | 284 (49.6) |
| ***Missing*** | 131 (14.5) | 16 (42.1) | 147 (25.6) |
| ***Serum Free Light Chain Ratio*** |  |  |  |
| ***Mean (SD)*** |  |  |  |
| ***Normal*** | 346 (64.7) | 17 (44.7) | 363 (63.4) |
| ***High*** | 58 (10.8) | 5 (13.2) | 63 (11) |
| ***Missing*** | 131 (24.5) | 16 (42.1) | 147 (25.6) |

IMD, Index of multiple deprivation; SD, standard deviation; NLR, neutrophil-to-lymphocyte ratio; eGFR, estimated glomerular filtration rate.

Supplementary Figure 1. Chi-squared goodness, AIC and BIC of fit for each latent class model, up to 10.


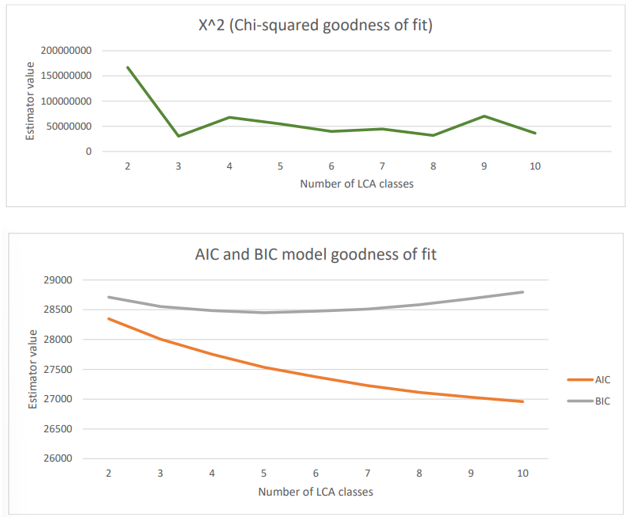


Supplementary Figure 2. Distribution of a) LCA and b) LASSO scores within the overall population.


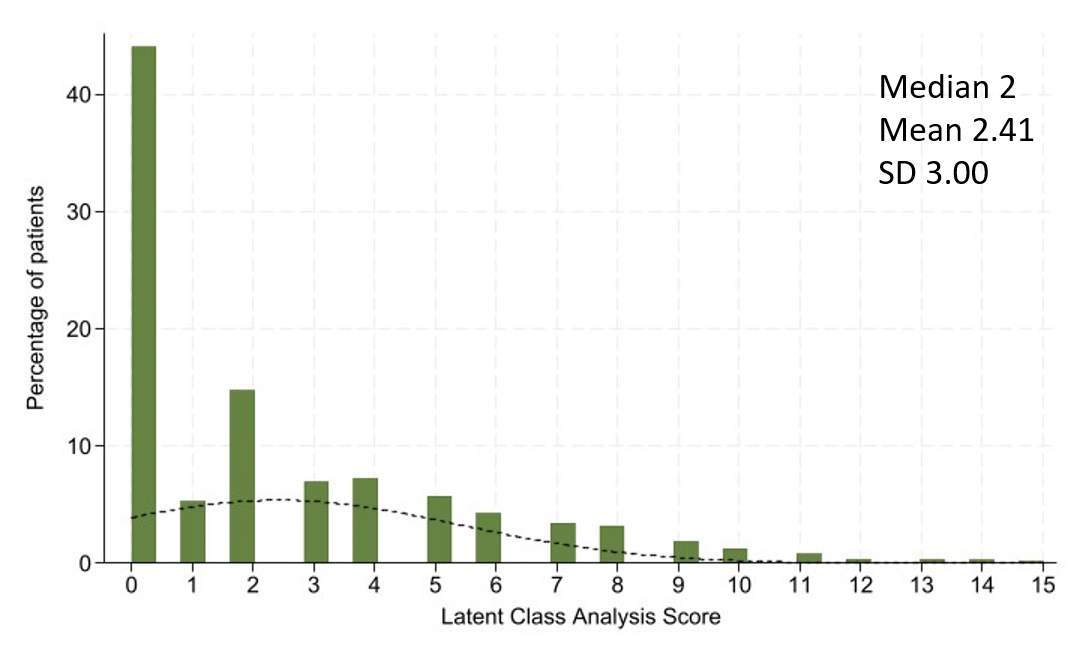


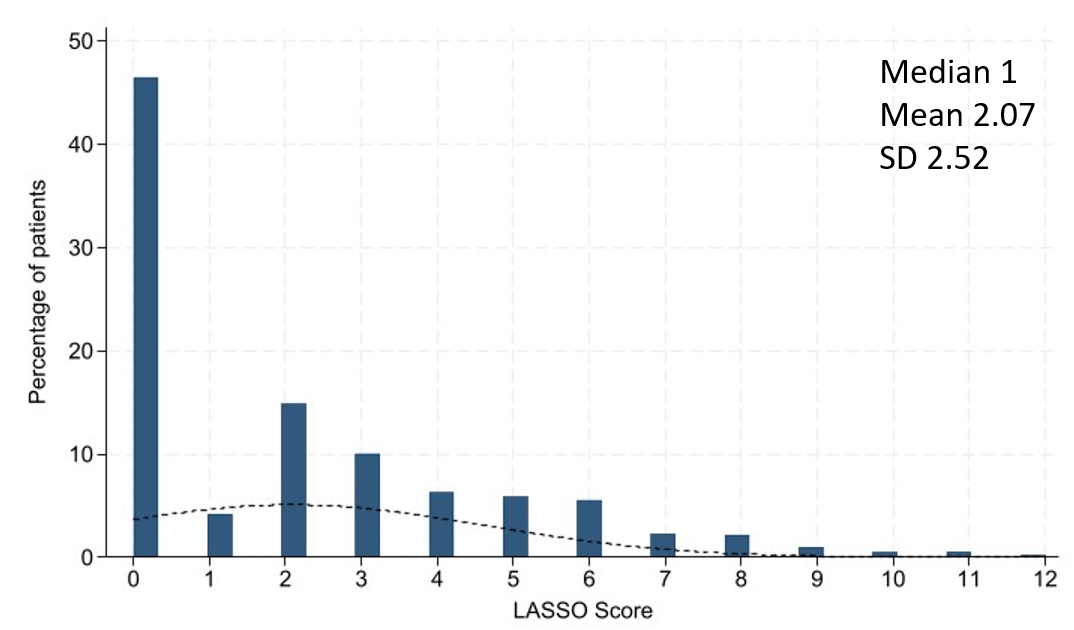

Supplement: online supplemental file 1 [file bmjopen-15-12-s001.docx]
