# Supplementary material for: Exploring a panel of serum biomarkers for cancer risk in patients with non-specific symptoms: a comparative analysis of feature selection methods
Source: BMJ Open. 2025 Dec 10;15(12):e099967. doi: 10.1136/bmjopen-2025-099967 (PMC12699618; doi:10.1136/bmjopen-2025-099967)
Supplement: Supplementary file 2 [file bmjopen-2025-099967.reviewer_comments.pdf]

PEER REVIEW HISTORY

BMJ Open publishes all reviews undertaken for accepted manuscripts. Reviewers are asked to complete a checklist review form and are provided with free text boxes to elaborate on their assessment. These free text comments are reproduced below.

ARTICLE DETAILS

Title (Provisional)

Exploring a panel of serum biomarkers for cancer risk in patients with non-specific symptoms: a comparative analysis of feature selection methods

Authors

Monroy-Iglesias, Maria Jose; Santaolalla, Aida; Martin, Sabine; North, Bernard; Moss, Charlotte Louise; Haire, Kate; Jones, Geraint; Steward, Lindsay; Cargaleiro, Carlos; Bruno, Flaminia; Millwaters, Juliet; Basyal, Chandra; Weild, Sarah; Russell, Beth; Van Hemelrijck, Mieke; Dolly, SO

VERSION 1 - REVIEW

**Reviewer**	**1**
**Name**	**Dack, Kyle**
**Affiliation**	**University of Bristol, MRC Integrative Epidemiology Unit**
**Date**	**12-Mar-2025**
**COI**	**None**

Statistical review summary:

The core statistical approach is reliable and it is an interesting paper, but there are a lot of important details missing from the methods. This harms the transparency of the paper and makes it hard to judge whether the two models have really been compared fairly or not. For example the timing of the measurements, biomarker measurement methods, and missing data handling.

The authors used the TRIPOD checklist but for many of the items marked as complete, unfortunately I do not think there is yet enough information (e.g. items 4b, 5a, 6, 7a, 9). Because of this I have added a lot of comments concerning the methods below – but the remainder of the paper has very few issues.

Abstract

1. Line 21. RDCs should not be plural because only one RDC is in the study.

2. Line 39. Guy's RDC is mentioned but was not yet introduced, and this will likely confuse some readers. I suggest either naming the RDC earlier in the abstract, or removing the name from this line.

3. Lines 41-51. The results are reported clearly. However, I suggest more clearly signposting that these are predictive modelling results. Currently the language used is similar to that used to standard causal modelling, and this could lead some readers to drawing inappropriate conclusions from the results. I think the authors could remedy this easy by just reminding readers occasionally that the model and results involve prediction and not inference, e.g. line 46: "A two point score increase predicted significantly higher odds of cancer diagnosis".

Introduction

4. Very well written and supported- no suggestions.

Methods

5. Line 17. Could the authors briefly explain why people may typically attend the RDC? This information will make the study population characteristics clearer.

6. The core of this study are the biomarkers, and as such there needs to be full details on when/how they were measured, procedures in the hospital and lab, and limits of detection. More guidance on the types of details to include can be found in the STROBE ME checklist, particularly item 4 described in this paper: https://core.ac.uk/reader/33035768?utm_source=linkout

7. With such a large number of biomarkers the authors may wish to put some details in a supplementary file rather than the main paper, but at least a high-level overview of the procedure should be available in the main paper.

8. Line 31. Similar to the above, please add more details of specifically what clinical and sociodemographic data was collected, including any transformations applied.

9. A subheading and paragraph to explain how the outcome was conceptualized and measured should be added – again the STROBE checklist or STROBE-ME checklist has helpful details explaining this.

10. At the moment the timings are not quite clear to me. Study data was collected between Dec 2016 and Sept 2023 – but is this for both biomarkers and cancer diagnosis, or do the date ranges differ? If someone attended the RDC in Sept 2023 and had biomarkers measured, would they have enough time for the diagnosis to be recorded and used in this study? Or did the authors apply exclusions or allow more time for outcome data collection? This could be explained when more details of the data collection are added.

11. Page 6 line 55. If PSA and CA125 were not part of the analysis, I don't think they should be described in the paper, unless there are some descriptive statistics relevant from these biomarkers?

12. Supplementary table 1. Clinical cutoff values are described, but are these values used in the analysis? Page 7 line 5 states that variables were kept continuous in the LASSO model – does this imply that for LCA the biomarkers were transformed to categories based on these clinical cutoffs? If so, please more directly state this in the text and explain why – doesn't it impact the comparability of the two models if variables are transformed only in one?

13. Page 7 line 35. Figure 1 stated that imputed data was used for the LASSO model, why couldn't this be used for the LCA model instead of only complete cases? Perhaps this would violate an assumption of the LCA model but please explain for any readers unfamiliar.

14. Page 7 line 44. Three model selection criteria were used – but AIC and BIC can often be in conflict, since BIC encourages a much more parsimonious model – was one criteria prioritised?

15. Page 8 line 11. Is it possible to include a profile of the missing data proportions as a supplementary file? Readers may want to know which biomarkers were impacted most. Please also add full details of the imputation process – software, package, any parameters.

16. Page 8 line 40. "LCA and LASSO scores were computed for each patient within each imputed dataset" – on page 7 and Figure 1 it states that only complete cases (no imputed data) were included in the LCA.

17. External validation – Please explain in more details how imputation was applied, and a brief summary of how exposure/outcomes were measured – e.g. "the procedure and timing of biomarker and cancer diagnosis measurements was broadly the same as in the primary data source".

Results

18. P10 line 17. This paragraph has been center-aligned.

19. Tables, figures, supplementary files – all very clear and well presented, no suggestions

20. The results are very clear and presented well. My only suggestion – for results and discussion - is the same as for the abstract results, that more reminders are given to readers that these are predictive modelling results which could be applied to future patients. E.g. P11 line 12 it would be more accurate to say both LCA and LASSO models predict significant increases in cancer risk. If more explanation is needed, this paper gives a good explanation for why different language should be used between predictive and inferential modelling https://www.stat.berkeley.edu/~aldous/157/Papers/shmueli.pdf

---

**Reviewer**	**2**
**Name**	**Tang, Hailin**
**Affiliation**	**Sun Yat-sen University Cancer Center, Department of Breast Oncology**
**Date**	**30-Aug-2025**

COI **None**

In this manuscript, the authors explored two statistical approaches—LCA and LASSO—to identify serum biomarkers associated with cancer risk in patients presenting with non-specific symptoms at Rapid Diagnostic Clinics. Based on these methods, the authors developed corresponding scoring systems and evaluated their predictive performance through logistic regression and decision curve analysis. The models are then externally validated using an independent RDC cohort, and the sensitivity and specificity of the two approaches were compared. The manuscript applies appropriate statistical methods with clear logic and clinically relevant objectives. However, several analytical and presentation issues remain to be clarified as follows.

Minor

1. The authors should clarify whether the imputation of missing data was performed exclusively for the LASSO analysis. This distinction would aid readers in understanding the methodological framework.

2. The scoring of LCA in the manuscript appears to rely solely on probability intervals, which may overlook the actual impact of individual variables on cancer risk. For instance, variables such as High Serum Kappa Light Chain show higher conditional item response probabilities in Class 2. Could this compromise their association with Class 3 and potentially reduce specificity? How did the authors balance sensitivity and specificity in this context?

3. Supplementary Table 2 has some formatting issues, including inconsistent font sizes and text in the second and third columns being partially hidden or blocked.

4. According to Table 2, the main differences between Class 3 and Class 2 appear to be concentrated in markers such as white blood cell count and neutrophils, which are almost entirely unhighlighted in Class 2. Although the unsupervised learning method fits the data well statistically, it may have led to an overlap in biomarkers between malignant and non-malignant conditions, thereby compromising interpretability and practical utility. It would be helpful if the authors could briefly describe the final diagnoses of samples in Class 3 and consider whether further stratification based on clinical interpretability is warranted. While the association with malignancy is stronger in Class 3, Class 2 also shows a significant correlation with cancer. Therefore, it would be useful to clarify how many malignant cases were assigned to Class 2 and Class 3, respectively. Regarding the biomarkers shared by both classes, it is not necessarily required to adjust their weights technically, but it would be appreciated if the authors could briefly discuss these issues.

5. Some conditional item response probabilities in Table 2 exceed the 0.4 threshold but are not highlighted.

6. It is recommended that the authors specify which dataset was used for the DCA. If the analysis was conducted on the training dataset, it would be valuable to include validation results using the external dataset.

VERSION 1 - AUTHOR RESPONSE

Reviewer #1 (Remarks to the author):

Abstract

1. Line 21. RDCs should not be plural because only one RDC is in the study.

2. Line 39. Guy's RDC is mentioned but was not yet introduced, and this will likely confuse some readers. I suggest either naming the RDC earlier in the abstract, or removing the name from this line.

3. Lines 41-51. The results are reported clearly. However, I suggest more clearly signposting that these are predictive modelling results. Currently the language used is similar to that used to standard causal modelling, and this could lead some readers to drawing inappropriate conclusions from the results. I think the authors could remedy this easy by just reminding readers occasionally that the model and results involve prediction and not inference, e.g. line 46: "A two point score increase predicted significantly higher odds of cancer diagnosis".

AUTHORS' REPLY: We thank the reviewer for these helpful comments regarding the abstract. We have made the following changes:

1. RDC terminology: The abstract has been updated to refer to a single Rapid Diagnostic Clinic (RDC) where appropriate.

2. Introduction of Guy's RDC: References to Guy's RDC in the Results section have been removed to avoid confusion, as the study setting is now introduced earlier in the abstract under the 'Setting' and 'Participants' sections.

3. Predictive language: We have revised the Results section to clearly emphasize that these are predictive modeling results rather than causal associations. Phrases such as "associated with" have been replaced with "predicted higher odds of cancer" and "predictors of cancer risk" to reinforce the predictive nature of the models.

Methods

5. Line 17. Could the authors briefly explain why people may typically attend the RDC? This information will make the study population characteristics clearer.

AUTHORS' REPLY: Thank you for this excellent suggestion. We completely agree that clarifying why patients typically attend the RDC helps contextualize our study population. We have amended the first paragraph of the Study Setting section to read as follows, providing the main reasons for referral to RDCs:

"Our study population comprised adult patients who attended Guy's RDC in Southeast London between December 2016 and September 2023. Patients are typically referred to the RDC by their general practitioner when presenting with non-specific symptoms, such as unexplained weight loss, fatigue, or persistent pain, that may be suggestive of an underlying malignancy but do not immediately point to a specific cancer site."

6. The core of this study are the biomarkers, and as such there needs to be full details on when/how they were measured, procedures in the hospital and lab, and limits of detection. More guidance on the types of details to include can be found in the STROBE ME checklist, particularly item 4 described in this paper: https://core.ac.uk/reader/33035768?utm_source=linkout

7. With such a large number of biomarkers the authors may wish to put some details in a supplementary file rather than the main paper, but at least a high-level overview of the procedure should be available in the main paper.

AUTHORS' REPLY: Thank you so much for this excellent and constructive feedback — we absolutely agree. We apologize for the oversight in not providing sufficient methodological detail on the biomarkers in the original submission. In response, we have now added information in the methods section describing how and when the biomarkers were ordered and measured, including that samples were taken at the first clinic visit and processed in the GSTT clinical biochemistry laboratory following standard hospital procedures. Serum and plasma were analysed using automated clinical chemistry platforms in accordance with laboratory protocols, with internal and external quality assurance procedures routinely followed.

Additionally, we have included Supplementary Table 1, which provides details on assay types, units, typical reference ranges, and detection limits for all biomarkers included in this study. We hope these additions now provide clear methodological transparency for readers while keeping the main text concise.

8. Line 31. Similar to the above, please add more details of specifically what clinical and sociodemographic data was collected, including any transformations applied.

AUTHORS' REPLY: Thank you again for this helpful comment. We have added more detail on the sociodemographic variables collected, including age, sex, ethnicity, and socioeconomic status (linked via postcode to the Index of Multiple Deprivation). Please see amended paragraph below:

"Clinical and sociodemographic data were collected retrospectively from the electronic patient records (EPR) for patients seen between December 2016 and May 2020. For patients seen from June 2020 onwards, data were collected prospectively and managed using REDCap electronic data capture tools hosted at GSTT (10). Variables included age, sex, ethnicity, and socioeconomic status, the latter obtained by linking patient postcodes to the Index of Multiple Deprivation using publicly available data from the Ministry of Housing, Communities & Local Government (2019). Lastly, patients who opted out of the NHS Digital National data opt out scheme were excluded from analysis (11). All data were collected and analysed under the ethical approval of GSTT Cancer Cohort (Ethics reference number: 23/NW/0105)."

9. A subheading and paragraph to explain how the outcome was conceptualized and measured should be added – again the STROBE checklist or STROBE-ME checklist has helpful details explaining this.

10. At the moment the timings are not quite clear to me. Study data was collected between Dec 2016 and Sept 2023 – but is this for both biomarkers and cancer diagnosis, or do the date ranges differ? If someone attended the RDC in Sept 2023 and had biomarkers measured, would they have enough time for the diagnosis to be recorded and used in this study? Or did the authors apply exclusions or allow more time for outcome data collection? This could be explained when more details of the data collection are added.

AUTHORS' RESPONSE: Thank you for these thoughtful comments. We have added a new Outcome Ascertainment section to clarify how the primary outcome was defined, measured, and timed. The NHS framework for rapid diagnostic clinics aims to provide a diagnosis within 28 days of referral. At Guy's RDC, this process can take additional weeks for some patients, but the median time to diagnosis remains approximately 28 days. Patients with suspicious imaging or clinical findings are referred to urgent site-specific pathways for confirmation, while others are discharged once malignancy has been reasonably excluded. Outcome data were collected until a formal diagnosis was recorded or the patient was discharged.

We have therefore included the following paragraph in the manuscript and hope that this provides sufficient clarity for readers:

"Outcome Ascertainment

The primary outcome of interest was a confirmed cancer diagnosis. Guy's RDC aims to provide a diagnosis within a few weeks of referral (median 28 days, Reference). Patients with suspicious imaging or clinical findings are referred to urgent site-specific pathways for confirmation, while others are discharged once malignancy has been excluded. Outcome data were collected until a formal diagnosis was recorded or the patient was discharged."

11. Page 6 line 55. If PSA and CA125 were not part of the analysis, I don't think they should be described in the paper, unless there are some descriptive statistics relevant from these biomarkers?

AUTHORS' REPLY: Thank you for this helpful comment. We agree that PSA and CA125 were not included in the analysis and do not contribute to the results presented. We had originally included them in the methods to acknowledge commonly measured cancer-specific markers, anticipating that readers might ask about their inclusion. However, since they were not analyzed and are not relevant to the study findings, we agree it is clearer to remove them from the list. The methods section has been updated accordingly to reflect only the biomarkers that were analyzed.

12. Supplementary table 1. Clinical cutoff values are described, but are these values used in the analysis? Page 7 line 5 states that variables were kept continuous in the LASSO model – does this imply that for LCA the biomarkers were transformed to categories based on these clinical cutoffs? If so, please more directly state this in the text and explain why – doesn't it impact the comparability of the two models if variables are transformed only in one?

AUTHORS' REPLY: Thank you for this thoughtful comment. We agree that it is important to clarify how biomarkers were handled in the two analyses. For the LASSO regression, biomarkers were kept as continuous variables to preserve the full range of information and ensure stable coefficient estimation, as dichotomizing continuous variables can distort effect estimates and reduce predictive performance. In contrast, for the LCA, biomarkers were transformed into binary variables using clinical cutoffs. This approach is common in LCA because using binary or categorical indicators facilitates model convergence and interpretability of the

resulting classes, whereas including many continuous variables can make class estimation more complex and harder to interpret clinically. To support the validity of this approach, we also performed a principal component analysis (PCA) using the continuous, standardized biomarkers prior to the main analyses. The resulting components were broadly consistent with the patterns observed in the LCA, providing reassurance that binarization did not fundamentally alter the underlying structure of the data. These PCA results are not included in the manuscript, as they were used only to inform our understanding of the data.

We have further clarified in the methods that biomarkers were handled differently for the two models, and added lines across the discussion acknowledging that this difference may limit direct comparability of effect estimates. We hope this explanation clarifies our approach and that you are satisfied with the rationale provided.

13. Page 7 line 35. Figure 1 stated that imputed data was used for the LASSO model, why couldn't this be used for the LCA model instead of only complete cases? Perhaps this would violate an assumption of the LCA model but please explain for any readers unfamiliar.

AUTHORS' REPLY: We thank the reviewer for this comment. LCA requires all included indicators to be observed for each patient, because the model estimates latent classes based on the patterns across these variables. Imputing missing biomarker values could interfere with this process, as standard imputation methods do not account for the underlying latent structure and could potentially bias the class assignments. For this reason, we restricted the LCA to patients with complete biomarker data. Importantly, the complete case subset was broadly similar to the full study population in terms of clinical and sociodemographic characteristics, suggesting that the use of complete cases did not meaningfully affect the representativeness of the results.

We agree that this reasoning was not clearly stated upfront, so we have edited our methods section to read as follows:

'We performed LCA, model-based clustering method that groups covariates into latent classes, capturing underlying unobservable conditions such as potential cancer (14). Given the criteria of LCA, which requires complete data on all included variables (in this case, biomarkers), we used a subset of patients with complete biomarker data for this analysis. Imputed values were not used, as standard imputation methods do not preserve the latent structure underlying LCA and could bias class formation. As previously mentioned, we used categorical variables based on standardized clinical cutoffs in our LCA.'

14. Page 7 line 44. Three model selection criteria were used – but AIC and BIC can often be in conflict, since BIC encourages a much more parsimonious model – was one criteria prioritised?

AUTHORS' REPLY: Thank you for this helpful comment. While AIC and BIC often guide LCA model selection, in our analysis the Chi-squared goodness-of-fit values showed the clearest indication of an optimal model, with a sharp improvement up to three classes and minimal change thereafter. The BIC curve also showed a modest elbow at three classes, consistent with the Chi-squared results, and a secondary one around five classes. We therefore selected the three-class solution based on the stronger inflection in the Chi-squared curve, its alignment with BIC, and its greater clinical interpretability.

Figure 1. Chi-squared goodness of fit for each latent class model, up to 10.

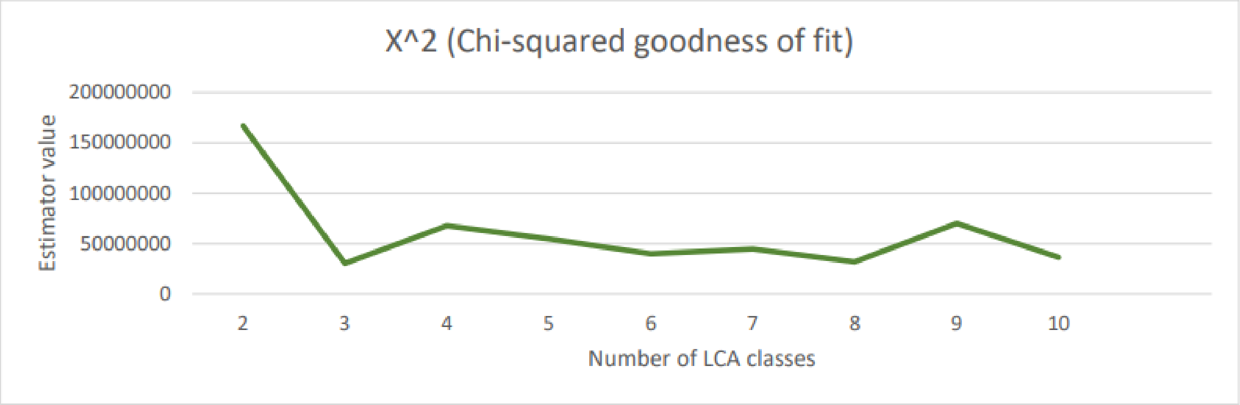

Figure 2. AIC and BIC for each latent class model, up to 10.

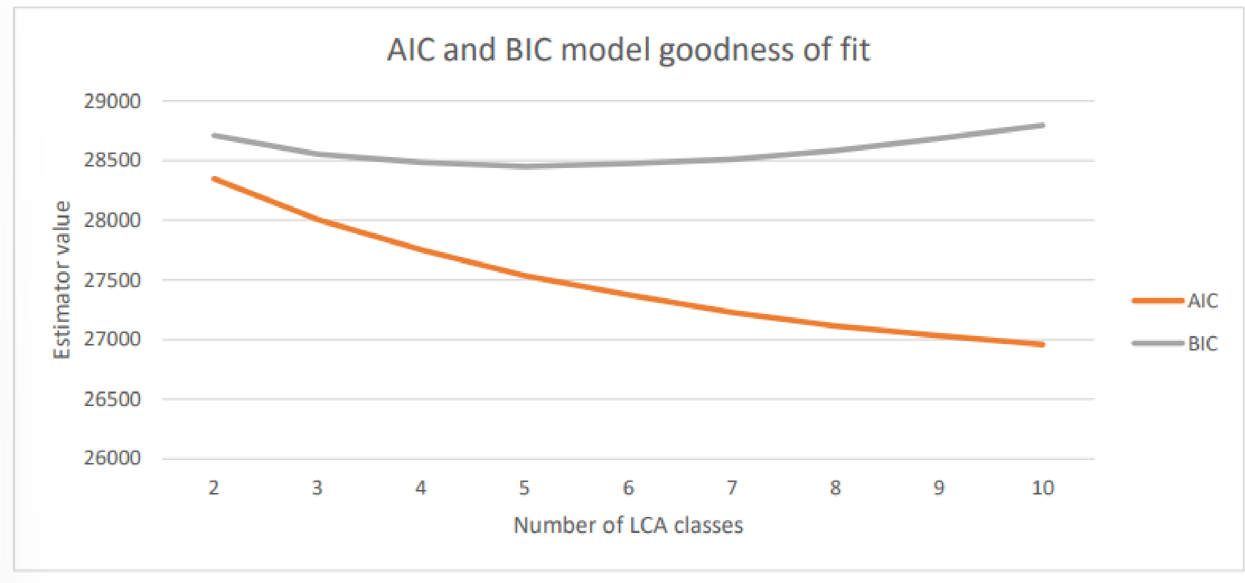

15. Page 8 line 11. Is it possible to include a profile of the missing data proportions as a supplementary file? Readers may want to know which biomarkers were impacted most. Please also add full details of the imputation process – software, package, any parameters.

AUTHORS' REPLY: Thank you for this helpful suggestion. We agree that providing more detail on the imputation process and the extent of missing data strengthens the transparency of our methods. We have therefore added a supplementary table (Supplementary Table 3) showing the proportion of missing data for each biomarker and expanded the Methods section to include additional details on the imputation process. Specifically, we now note that multiple imputation by chained equations (MICE) was performed in R (package

mice), using predictive mean matching for continuous variables and logistic regression for binary variables, with 20 iterations per imputation. The revised paragraph in the Methods now reads as follows:

"Missing data

Among the predictor variables, the proportion of missing data ranged from 0% to 53% (Supplementary Table 3). Missing biomarker data were handled using multiple imputation by chained equations (MICE) in R (package mice) under the 'missing at random' assumption. Predictive mean matching was applied for continuous variables and logistic regression for binary variables, with 20 iterations per imputation cycle. Two imputation models were created, each generating ten imputed datasets and including all predictor variables, endpoint indicators (i.e., cancer diagnosis), sex, and age. These imputed datasets were used for all subsequent analyses."

16. Page 8 line 40. "LCA and LASSO scores were computed for each patient within each imputed dataset" – on page 7 and Figure 1 it states that only complete cases (no imputed data) were included in the LCA.

AUTHORS' REPLY: We thank the reviewer for noting this point. We have clarified in the manuscript that the LCA was performed using complete cases only, as the goal at that stage was to identify latent biomarker patterns for variable selection without introducing imputation-related bias. Multiple imputation was then used for subsequent analyses, including LASSO selection and logistic regression modeling, where the derived scores were applied across the imputed datasets. The text has been revised for consistency:

"Missing data

Among the predictor variables, the proportion of missing data ranged from 0% to 53% (Supplementary Table 3). Missing biomarker data were handled using multiple imputation by chained equations (MICE) in R (package mice) under the 'missing at random' assumption. Predictive mean matching was applied for continuous variables and logistic regression for binary variables, with 20 iterations per imputation cycle. Two imputation models were created, each generating ten imputed datasets and including all predictor variables, endpoint indicators (i.e., cancer diagnosis), sex, and age. These imputed datasets were used for subsequent analyses, including LASSO selection and logistic regression, while LCA was performed solely for variable selection using complete cases."

"Logistic Regression for scores

Once the LCA and LASSO scores were derived, they were applied to each imputed dataset and analysed as continuous and binary variables, using a cutoff determined based on the score distribution in the overall population. Binary logistic regression was then employed to assess the association between these scores and overall cancer risk. Both unadjusted and adjusted models were considered, with sex and age as covariates. Rubin's rules were used to combine estimates and standard errors across imputations, resulting in a pooled model for predictor selection."

17. External validation – Please explain in more details how imputation was applied, and a brief summary of how exposure/outcomes were measured – e.g. "the procedure and timing of biomarker and cancer diagnosis measurements was broadly the same as in the primary data source".

AUTHORS' REPLY: We thank the reviewer for highlighting the need for additional detail regarding the external validation. We have clarified in the manuscript that the Queen Elizabeth Hospital RDC (QEH RDC) was established after Guy's RDC in September 2022 and operated similarly, following the same referral patterns, with biomarkers collected at the first RDC visit. We also specified that the validation process applied the previously developed LCA and LASSO scores to the external dataset, using the same multiple imputation procedure (MICE with the same parameters as Guy's RDC) and subsequent logistic regression.

Results

18. P10 line 17. This paragraph has been center-aligned.

AUTHORS' REPLY: We thank the reviewer for the observation, we have aligned the whole manuscript to the left.

19. Tables, figures, supplementary files – all very clear and well presented, no suggestions

20. The results are very clear and presented well. My only suggestion – for results and discussion - is the same as for the abstract results, that more reminders are given to readers that these are predictive modelling results which could be applied to future patients. E.g. P11 line 12 it would be more accurate to say both LCA and LASSO models predict significant increases in cancer risk. If more explanation is needed, this paper gives a good explanation for why different language should be used between predictive and inferential modelling https://www.stat.berkeley.edu/~aldous/157/Papers/shmueli.pdf

AUTHORS' REPLY: We thank the reviewer for this helpful suggestion. We have carefully reviewed both the Results and Discussion sections and amended the wording to emphasize that our findings reflect predictive modeling rather than causal inference. Throughout these sections, we now clarify that the LCA and LASSO scores predict higher odds of cancer diagnosis rather than implying risk or causal effects. We hope these changes adequately address the reviewer's concerns and improve clarity for readers regarding the predictive nature of our analyses.

Reviewer #2 (Remarks to the author):

1. The authors should clarify whether the imputation of missing data was performed exclusively for the LASSO analysis. This distinction would aid readers in understanding the methodological framework.

AUTHORS' REPLY: We thank the reviewer for this helpful suggestion. We have clarified in the manuscript that multiple imputation was not performed for the LCA variable selection, which was restricted to complete cases for variable selection, but was applied for subsequent analyses, including LASSO and logistic regression for estimates associated with the scores. We hope that the added details in the revised methods section (please see below) make this distinction clear and improve the readability of our methodological framework.

"Missing data

Among the predictor variables, the proportion of missing data ranged from 0% to 53% (Supplementary Table 3). Missing biomarker data were handled using multiple imputation by chained equations (MICE) in R (package mice) under the 'missing at random' assumption. Predictive mean matching was applied for continuous variables and logistic regression for binary variables, with 20 iterations per imputation cycle. Two imputation models were created, each generating ten imputed datasets and including all predictor variables, endpoint indicators (i.e., cancer diagnosis), sex, and age. These imputed datasets were used for subsequent analyses, including LASSO selection and logistic regression, while LCA was performed solely for variable selection using complete cases."

2. The scoring of LCA in the manuscript appears to rely solely on probability intervals, which may overlook the actual impact of individual variables on cancer risk. For instance, variables such as High Serum Kappa Light Chain show higher conditional item response probabilities in Class 2. Could this compromise their association

with Class 3 and potentially reduce specificity? How did the authors balance sensitivity and specificity in this context?

AUTHORS' REPLY: We thank the reviewer for this insightful comment. Some biomarkers, such as high SKLC, are elevated in both Class 2 and Class 3, reflecting their role in systemic inflammation and in a subset of pre-malignant conditions (e.g., MGUS). The LCA score gives higher weight to markers enriched in Class 3, which is most strongly associated with malignancy. While this overlap may slightly reduce specificity, the scoring system intentionally prioritizes sensitivity, as identifying potential cancer cases is the key clinical goal in the NSS setting. This approach balances sensitivity and specificity, allowing the score to capture patients at highest risk while maintaining clinical utility.

3. Supplementary Table 2 has some formatting issues, including inconsistent font sizes and text in the second and third columns being partially hidden or blocked.

AUTHORS' REPLY: We thank the reviewer for pointing out the formatting issues in Supplementary Table 2. The table has been corrected to ensure consistent font sizes and that all text in each column is fully visible.

4. According to Table 2, the main differences between Class 3 and Class 2 appear to be concentrated in markers such as white blood cell count and neutrophils, which are almost entirely unhighlighted in Class

AUTHORS' REPLY: We thank the reviewers for making note of this oversight. We have now highlighted all biomarkers above 0.40.

5. Although the unsupervised learning method fits the data well statistically, it may have led to an overlap in biomarkers between malignant and non-malignant conditions, thereby compromising interpretability and practical utility. It would be helpful if the authors could briefly describe the final diagnoses of samples in Class 3 and consider whether further stratification based on clinical interpretability is warranted. While the association with malignancy is stronger in Class 3, Class 2 also shows a significant correlation with cancer. Therefore, it would be useful to clarify how many malignant cases were assigned to Class 2 and Class 3, respectively. Regarding the biomarkers shared by both classes, it is not necessarily required to adjust their weights technically, but it would be appreciated if the authors could briefly discuss these issues.

AUTHORS’ REPLY: We thank the reviewer for this suggestion. We have amended the Results section to increase transparency regarding the distribution of malignancy across LCA classes. Specifically, we now report that both Class 3 (“very serious condition”) and Class 2 (“serious condition”) were associated with significantly higher odds of cancer compared to Class 1 (“Normal”) (OR 4.26; 95% CI 2.26–8.02 and OR 2.88; 95% CI 1.78–4.45, respectively) (Supplementary Table 6). Examination of the final diagnoses showed that 15% of individuals in Class 3 had a malignant condition, compared to 10% in Class 2 (Supplementary Table 7). Accordingly, Class 3 was prioritized for biomarker selection due to its higher enrichment for malignancy.

To address the reviewer’s comments on biomarker overlap and interpretability, we also added the following text to the discussion:

“Within the LCA, some biomarkers—such as WCC and neutrophils—were elevated in both Class 2 and Class 3, reflecting the continuum of serious conditions captured by this unsupervised method. Class 3 was enriched for malignancy, supporting its prioritization for biomarker selection to focus on features most indicative of cancer, while the overlap with Class 2 does not compromise the clinical utility of the score.”

We hope that these changes clarify the distribution of cancer cases across classes and provide context for the selection of class 3 for biomarker scoring.

5. Some conditional item response probabilities in Table 2 exceed the 0.4 threshold but are not highlighted.

AUTHORS’ REPLY: We thank the reviewer for noting this. The missing conditional item response probability for creatinine has now been highlighted in Table 2 to ensure consistency with our 0.4 threshold.

6. It is recommended that the authors specify which dataset was used for the DCA. If the analysis was conducted on the training dataset, it would be valuable to include validation results using the external dataset.

We thank the reviewer for this helpful suggestion. The DCA was conducted on the primary study dataset to illustrate the clinical utility of the binary LCA and LASSO scores across a range of risk thresholds. Unfortunately, we no longer have access to all required variables in the external validation dataset to perform DCA in that cohort. Nevertheless, external validation already confirms that higher scores are associated with increased cancer risk, supporting the clinical relevance and generalizability of the scoring systems. We agree that future studies applying DCA in independent cohorts would be valuable to further assess clinical utility.

VERSION 2 - REVIEW

Reviewer 2

Name Tang, Hailin

Affiliation Sun Yat-sen University Cancer Center, Department of Breast Oncology

Date 22-Nov-2025

COI

There are no further comments.
